# Insights into digit evolution from a fate map study of the forearm using *Chameleon*, a new transgenic chicken line

**DOI:** 10.1242/dev.202340

**Published:** 2024-06-28

**Authors:** Julia Dong Hwa Oh, Lu Freem, Dillan D. Z. Saunders, Lynn McTeir, Hazel Gilhooley, Melany Jackson, James D. Glover, Jonathan Smith, Jeffrey J. Schoenebeck, Laura A. Lettice, Helen M. Sang, Megan G. Davey

**Affiliations:** ^1^ Functional Genetics, The Roslin Institute R(D)SVS, CMVM, University of Edinburgh, Edinburgh EH25 9RG, UK; ^2^ Genetics and Genomics, The Roslin Institute R(D)SVS, CMVM, University of Edinburgh, Edinburgh EH25 9RG, UK; ^3^ MRC Human Genetics Unit, Institute of Genetics and Cancer, University of Edinburgh, Edinburgh EH4 2XU, UK

**Keywords:** Fate map, Ulna, Evolution, SHH, Limb, Transgenic chicken

## Abstract

The cellular and genetic networks that contribute to the development of the zeugopod (radius and ulna of the forearm, tibia and fibula of the leg) are not well understood, although these bones are susceptible to loss in congenital human syndromes and to the action of teratogens such as thalidomide. Using a new fate-mapping approach with the *Chameleon* transgenic chicken line, we show that there is a small contribution of *SHH*-expressing cells to the posterior ulna, posterior carpals and digit 3. We establish that although the majority of the ulna develops in response to paracrine SHH signalling in both the chicken and mouse, there are differences in the contribution of *SHH*-expressing cells between mouse and chicken as well as between the chicken ulna and fibula. This is evidence that, although zeugopod bones are clearly homologous according to the fossil record, the gene regulatory networks that contribute to their development and evolution are not fixed.

## Teaser

**Summary:** The widely held assumption that the ulna remains unchanged during limb evolution has overlooked the evidence that the gene regulatory networks involved are not static.

## INTRODUCTION

**Fig. 1.**
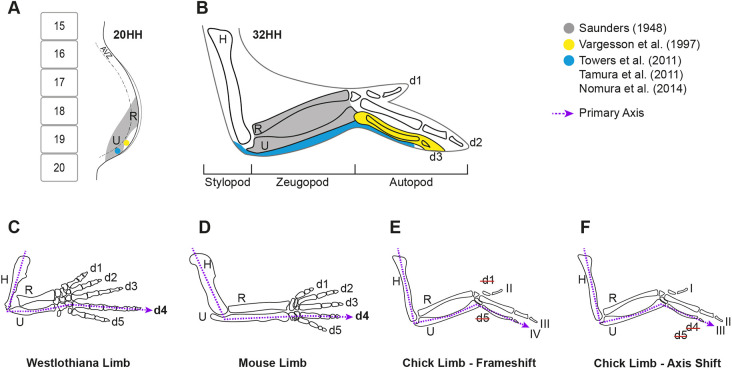
**Summary of published fate maps and hypotheses for digit loss in chicken.** (A,B) Amalgamation of previous fate map studies of the chick wing in the stage 20 HH chick wing bud with somites and avascular zone as reference (A) and in the stage 32 HH chick wing (B). Grey shading is derived from Saunders (1948) and yellow shading from Vargesson et al. (1997). Blue shading show an agreement of results from Towers et al. (2011), Tamura et al. (2011) and Nomura et al. (2014). (C,D) Diagrams of the primary axis represented by a purple dotted line going through the humerus, ulna and digit 4 in the Westlothiana limb (C) and mouse limb (D). (E) Schematic of the frameshift hypothesis, in which the primary axis continues to course through the ulna and digit 4 in the chick wing, with a loss of digits 1 and 5. (F) Schematic of the axis shift hypothesis, in which the primary axis has shifted and now courses through digit 3 (III) in the chick wing. HH, Hamburger Hamilton stage; AVZ, avascular zone; H, humerus; R, radius; U, ulna; d, digit.

Limbs first form as small paired forelimb or hindlimb buds growing from the flank of a developing embryo (Tickle, 2015). The mesodermal cell component of the limb bud, derived from the lateral plate mesoderm (Gros and Tabin, 2014), forms the majority of the limb skeleton from the proximal shoulder/pelvic girdle to the digit tips. The cells that make up the early limb bud look homogenous but fate maps of the stage 20 HH (Hamburger and Hamilton, 1951) chicken limb show that specific areas of mesoderm are already specified to form the shoulder/pelvic girdle, stylopod (humerus/femur) or zeugopod (radius and ulna, tibia/fibula), and by 24 HH, the autopod (digits; Dudley et al., 2002; Nomura et al., 2014; Sato et al., 2007; Saunders, 1948; Vargesson et al., 1997; Fig. 1A,B). Within the autopod, the origin, number and signalling pathways that pattern the antero-posterior identity of digits have been well studied (Harfe et al., 2004; Tamura et al., 2011; Towers et al., 2008, 2011; Zhu et al., 2022). Although the specification of the zeugopod region within the proximo-distal axis of limb bud has been also been examined (Dudley et al., 2002; McCusker and Rosello-Diez, 2022; Rosello-Diez et al., 2011; Sato et al., 2007), how two bones with different antero-posterior identities, the anterior radius and posterior ulna, develop from this area has not been thoroughly investigated. Human conditions in which either the radius or ulna are lost highlight the separate identities of these bones; unlike radial deficiency, ulnar deficiency is rarely associated with systemic syndromes (Bednar et al., 2009) and radial deficiency is more common than ulnar deficiency, including in thalidomide cases (Vargesson, 2019).

The zeugopod is subject to many of the same patterning mechanisms as the autopod and parallels between these parts of the limb can be drawn, specifically the action of the Sonic hedgehog (SHH) pathway on the development of the antero-posterior axis (Chiang et al., 2001). In the chicken wing, *SHH* is expressed in the mesoderm-derived organiser of the limb, the ‘zone of polarising activity’ (ZPA), from stage 18 HH, and it is thought that the relative balance of paracrine and autocrine SHH signalling, along with cell proliferation, is central to establishing both digit number and identity (Towers et al., 2008; Zhu et al., 2022). In human and mouse, a loss of SHH signalling causes a loss of posterior digit identity and dysplastic zeugopod formation (Ianakiev et al., 2001; Zhu et al., 2008), demonstrating that SHH signalling is required for posterior limb identity in both the autopod and zeugopod. Although in human and mouse it is difficult to ascertain the identity of any forearm elements (Ianakiev et al., 2001; Zhu et al., 2008), in the chicken OZD mutant, which lacks *SHH* expression in the limb buds, the anterior bones of the zeugopod, the radius and tibia, remain, while the posterior bones of the zeugopod, the ulna and fibula, are lost, suggesting that, at least in chicken, the ulna and fibula are dependent on SHH signalling (Ros et al., 2003).

There is also a distinction in the limb between skeletal elements that derive from cells expressing *Shh/SHH* within the ZPA (and therefore subject to short-range autocrine Shh/SHH signalling) and those skeletal elements that receiving long-range paracrine Shh/SHH signals from ZPA cells. In the mouse, *Shh*-expressing cells from the ZPA contribute primarily to digits 4 and 5 in the forelimb and hindlimb (Harfe et al., 2004; Scherz et al., 2007; Zhu et al., 2022) as well as making a small contribution to digit 3 and the ulna (Harfe et al., 2004; Scherz et al., 2007), indicating that part of the ulna is subject to both autocrine and paracrine Shh signalling (Ahn and Joyner, 2004). *SHH*-expressing cells do not contribute to any of the three digits of the chicken wing (Towers et al., 2011). This has been used as evidence to determine which two digits birds lost during evolution from the pentadactyl limb (Tamura et al., 2011; Towers et al., 2011; Xu and Mackem, 2013), an important paradigm in the study of evolutionary development.

The evolution of the bird wing, in particular understanding which two digits were ‘lost’ and which three remain in the modern tridactyly wing, is studied both to understand the context of the bird wing as a model of vertebrate limb development and morphological evolution (Brusatte, 2017; Richardson et al., 2009). The focus on the majority of the research in this area has been to understand which of the three bird digits are homologous to the five of a pentadactyl limb, such as a mouse, human or basal archosaur, an example of which is the basal tetrapod (e.g. Westlothiana; Smithson et al., 2011; Fig. 1C), from which all vertebrate limb patterns arose. There are conflicting interpretations of digit homology due to an incomplete fossil record, which is further confounded by ambiguity in assigning a universal digit identity to the three bird digits using either adult or embryological data (Burke and Feduccia, 1997; Chatterjee, 1998; de Bakker et al., 2013, 2021; Hinchliffe and Hecht, 1984; Kawahata et al., 2019; Larsson and Wagner, 2002; Richardson, 2012; Salinas-Saavedra et al., 2014; Stewart et al., 2019; Tamura et al., 2011; Towers, 2018; Towers et al., 2008, 2011; Vargas and Fallon, 2005; Welten et al., 2005; Woltering and Duboule, 2010; Xu and Mackem, 2013; Xu et al., 2014). In these studies, evolutionary anatomical changes in the zeugopod bones have been overlooked, as homology of the radius and ulna is easily assigned and both are clearly present throughout the fossil record. Rather, the emphasis has been that morphology of the carpals and digits has evolved distal to the ‘unchanging’ bony anatomy of the forearm: the radius and ulna (Fig. 1C,D). This is embodied in a foundation principle, the ‘primary limb axis’ hypothesis (Salinas-Saavedra et al., 2014; Shubin and Alberch, 1986), which emphasises the line of conserved morphology that includes the humerus and ulna around which distally digits have evolved. How palaeontological, anatomical and embryological data have been interpreted has led to the development of the ‘frame-shift’ and ‘axis-shift’ hypotheses (Xu and Mackem, 2013).

The ‘frame-shift’ model (Fig. 1E), primarily based on embryological evidence such as the development of SOX9^+^ digit primordia, proposes that the primary axis is maintained and the ulna-digit 4 articulation remains unchanged, but that a modified digit 4 takes on a morphological identity of a digit III through a homeotic transformation, thereby concluding that digit 1 and 5 are lost (de Bakker et al., 2013; Tamura et al., 2011). Alternatively, based on both fossil and embryological data, the contribution of *SHH*-expressing cells to the digits as an indicator of lineage, the ‘axis-shift’ model (Fig. 1F) suggests that the articulation between the primary axis/ulna shifts from digit 4 to digit 3, but this does not account for how the change in this relationship might have occurred (Towers et al., 2011). A limitation of all these studies has been a lack of analysis of the bones proximal to the digits, although analysis of the carpals suggests that these bones, articulating the zeugopod with the autopod, have been even more radically altered than the digits (Botelho et al., 2014). The contribution of *SHH*-expressing cells to the ulna of the chick has not been described, nor has the contribution of *Shh/SHH*-expressing cells to the mouse or chicken fibula; therefore, a universal theory of the contribution of SHH cells to the zeugopod comes only from limited analysis of the mouse forelimb.

We propose that understanding developmental events that pattern the limb proximal to the digits, including the contribution of *SHH*-expressing cells to elements of the posterior bird forelimb and carpals, is central to understanding the evolution of the avian primary limb axis and digits that articulate with it. We therefore sought to identify the exact location of the ulna anlage and explore its relation to the ZPA using a new anatomical approach to fate mapping with the *Chameleon* transgenic chicken line. We show that *SHH*-expressing ZPA cells contribute to the carpals and digit 3 cartilage in a developmental stage-dependent manner similar to the mouse, demonstrating an embryological relationship between these skeletal elements, but that the ZPA contribution between the chick ulna, the mouse ulna and chick fibula are variable.

## RESULTS

### Applying TAT-Cre to the Chameleon chicken line as a novel fate mapping technique

**Fig. 2.**
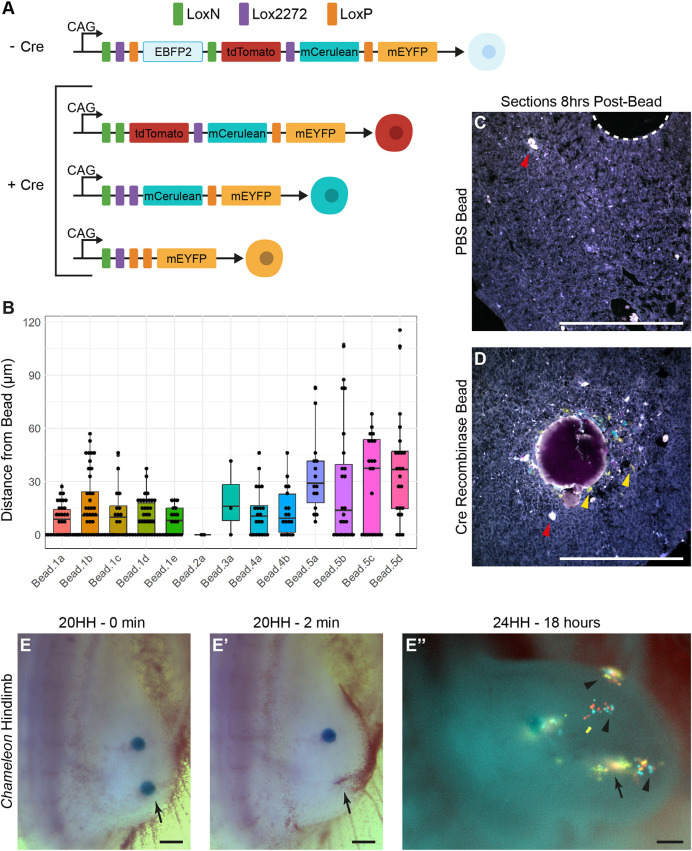
**Cytbow construct and parameters for inducing fluorescence with TAT-Cre.** (A) A schematic of the Cytbow transgene that ubiquitously expresses EBFP but with added Cre-recombinase undergoes recombination to express one of tdTomato, mCerulean or mEYFP. (B) Box plot showing the distances at which fluorescent cells were measured from the bead surface 8 h after Tat-Cre bead insertion. Individual points represent the distance of individual cells from the bead surface; the box outlines the interquartile range, including median; whiskers indicate 1.5 interquartile range above the third and first quartile; points beyond the whiskers are outliers. (C,D) Sections of limbs show no fluorescence with a control bead (*n*=3), as outlined with a white dashed line (C), versus multiple fluorescent cells 8 h post-insertion of a bead soaked in TAT-Cre recombinase (D) (*n*=3). Red arrowheads indicate auto-fluorescent blood cells that appear white when channels are merged; yellow arrowheads indicate examples of fluorescent cells, which are labelled in light blue, yellow or pink. (E-E″) Two TAT-Cre-soaked beads inserted simultaneously in a 20 HH hindlimb bud (E), one of which was removed after 2 min (arrow in E′) and imaged again live after 18 h (E″) (*n*=4). Similar levels of fluorescence were observed even after bead removal (arrow in E″). Black arrowheads indicate ectoderm labelling. HH, Hamburger Hamilton stage. Scale bars: 200 µm.

Fate mapping approaches using the chicken embryo have been fundamental in understanding vertebrate limb development, as well as the development of the neural crest and other developing systems (Yokota et al., 2011; Davey et al., 2018). To improve upon previous cell labelling approaches, with the aim of producing transgenic embryos capable of stably expressing fluorescent proteins in clones *in ovo* through electroporation approaches, we produced a germline transgenic chicken line *Chameleon* containing a single insertion of the Cytbow transgene carried by a Tol2 transposon vector (Fig. 2A, Figs S1, S2). This is a fluorescent reporter expression construct, which ubiquitously expresses H2B-EBFP2, with the potential to instead express one of three fluorescent proteins, tdTomato, mCerulean or mEYFP (tdTom, mCeru and mEYFP, respectively), upon Cre recombinase-mediated recombination (Loulier et al., 2014; Fig. 2A, Fig. S1). Although induction of recombination and subsequent expression of tdTom, mCeru or mEYFP in other vertebrate systems is routinely mediated via expression of Cre-recombinase in a tissue-specific manner (Livet et al., 2007), we realised that, in addition to electroporation of Cre-expressing constructs, topical application of the cell permeable TAT-Cre recombinase (TAT-Cre) could provide an alternative approach to induce stable expression of tdTom/mCeru/mEYFP in localised areas of the chicken embryo, providing a useful tool to fate map organs and tissues for which there are no tissue-specific Cre recombinase-expressing lines, or tissues that do not have any known tissue-specific promoters. On testing the methods by which topically applied TAT-Cre could be applied *in vitro* and *in vivo*, and the subsequent dynamics of tdTom/mCeru/mEYFP expression (Fig. S3-S8, see supplementary Materials and Methods for further details), we have found in the limb bud that topical application of TAT-Cre using Affi-Gel Blue beads results in regionally and temporally limited recombination and tdTom/mCeru/mEYFP expression, enabling permanent labelling of small discrete areas of the limb bud mesenchyme and determination of their subsequent developmental fate. Live embryo imaging determined that induction of tdTom/mCeru/mEYFP can be observed between 2 and 5 h after application of TAT-Cre, dependent on the visualisation approach, and that all three induced fluorophores are equally expressed (Fig. S3). In a stage 20 HH limb, 8 h after application of TAT-Cre via an 80-100 µm Affi-Gel Blue bead, 34% of tdTom/mCeru/mEYFP cells remained in contact with the bead surface, and cells not touching the bead were an average of 25 µm from the bead surface, with a maximal range of 115 µm from the bead (Fig. 2B-D), indicating that TAT-Cre action, leading to recombination and tdTom/mCeru/mEYFP expression, occurs primarily within the immediate location of the bead in the limb mesenchyme. In addition, in the limb mesenchyme and in early chick (EC) culture we have found action of TAT-Cre is both transient and temperature dependent. Application in embryos at room temperate does not induce tdTom/mCeru/mEYFP expression, even if subsequently incubated at 38°C. Induction of tdTom/mCeru/mEYFP in areas in which beads are placed and immediately removed, compared with beads left in place, is similar, suggesting that the action of TAT-Cre is immediate and transient (Fig. 2E), and is unlikely to continue to induce recombination and tdTom/mCeru/mEYFP expression for a prolonged period after application. The duration and distance from the bead of cells newly expressing tdTom/mCeru/mEYFP differs between ex-ovo EC culture (Fig. S5-S8) and limb bud application; we hypothesise that this is due to excess TAT-Cre protein transferring and labelling the limb bud ectoderm (arrowheads Fig. 2E″) during bead placement in limb bud mesenchyme, limiting excess TAT-Cre protein transfer to mesenchyme during application. We suggest when using topically applied TAT-Cre, researchers examine the dynamics TAT-Cre action in their tissue of interest.

### The ulna arises from a discrete area within the chick limb bud

**Fig. 3.**
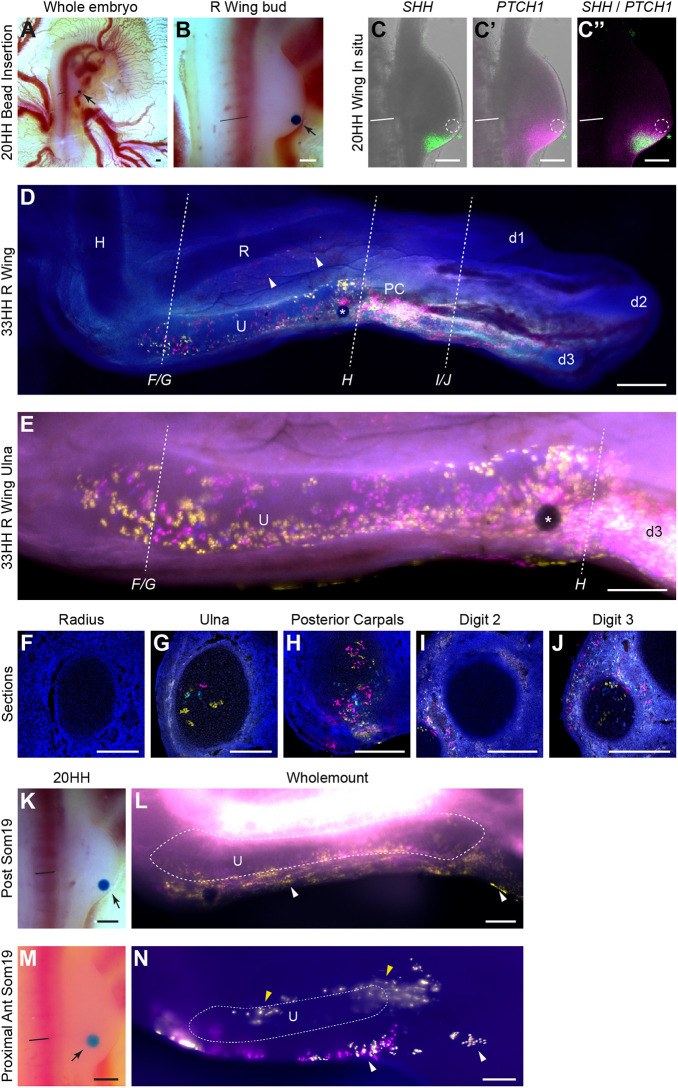
**Fate map of chick ulna using *Chameleon*.** (A,B) Placement of beads soaked in TAT-Cre (arrow) that maps the ulna in stage 20 HH *Chameleon* chick wing buds shown in whole embryo (A) and at higher magnification (B). Straight black or white lines indicate the anterior-most edge of somite 19. (C-C″) Stage 20 HH chick wing buds with inert bead (dashed white circle) inserted in the anterior half of somite 19 (*n*=3) (as in B) followed by HCR *in situ* hybridisation with *SHH* (C) and *PTCH1* (C′) both shown in brightfield. (C″) Merge of *SHH* and *PTCH1*. Asterisks indicate the anterior-most edge of the ZPA. Straight white lines indicate the anterior-most edge of somite 19. (D) *Chameleon* stage 33 HH chick wing showing fluorescent cells (magenta, yellow and cyan with white indicating overlap) in the ulna and digit 3 (*n*=3), which were recombined on exposure to TAT-Cre delivered by bead (as in B). White arrowheads indicate experimental artefacts. (E) Close-up of D with focus on the ulna. White asterisks indicate the location of the bead. (F-J) Cross-sections of radius (F), ulna (G), posterior carpals (H), digit 2 (I) and digit 3 (J) taken at the levels indicated by dashed lines in D and E. (K-M) TAT-Cre soaked beads placed adjacent to the posterior somite 19 in 20 HH *Chameleon* chick wings (arrow, K) will not give rise to the ulna (L) while adjacent to anterior somite 19; beads placed more proximally (arrow, M) will also not give rise to the ulna (N) (*n*=5). White arrowheads in L and N indicate ectoderm labelling; yellow arrowheads indicate muscle labelling. HH, Hamburger Hamilton stage; H, humerus; R, radius; U, ulna; PC, posterior carpals; d1-d3, digits 1-3. Scale bars: 200 µm.

The precise origin of the presumptive zeugopod has not been extensively investigated in mouse or chicken due to the technical limitations of other fate-mapping approaches. Using *Chameleon* embryos, we examined the origin of the ulna from within the presumptive zeugopod-forming region at 20 HH, as identified by Saunders (Saunders, 1948; Fig. 1A,B). With Saunders' map as a guide, beads soaked in TAT-Cre were inserted around the ulnar area of the presumptive zeugopod-forming region in 20 HH *Chameleon* chicken limb buds, to determine that the ulna arises from a highly discrete area of cells in the distal limb bud, parallel to the anterior half of somite 19 (Fig. 3A,B). This region of the limb lies above the *SHH*-expressing ZPA cells but expresses *PTCH1*, a hedgehog receptor whose expression is induced by the ligand, demonstrating that the area is subject to paracrine SHH signalling (Fig. 3C,C″). Stage 33 HH wings were subsequently analysed for anatomical distribution of fluorescent cells, which were found to be located in the ulna, posterior carpals and digit 3 (Fig. 3D-J). Beads placed either more proximally or within the ZPA parallel to the posterior half of somite 19, which expresses both *SHH* and *PTCH1*, did not result in fluorescent labelling of the ulna (Fig. 3K-N).

Closer analysis of limbs labelled in the anterior half of somite 19 (*SHH*^−^/*PTCH1*^+^) demonstrated that fluorescent cells spanned the length of the ulna (Fig. 3D-G) and, remarkably, were largely contained within the ulna cartilage (Fig. 3E-G), indicating that cells within 50 µm of the bead at stage 20 HH contributed to the entire length of the ulna. Sections showed no labelled cells in the radius (Fig. 3F) and few cells in the ulnar perichondrium or adjacent soft tissues (*n*=3/3, Fig. 3E-G). In addition to the ulna, the cartilage of posterior carpals (Fig. 3H) and the cartilage of digit 3 (Fig. 3J) also contained fluorescent cells, as well as soft tissue adjacent to the cartilage of digit 2 (Fig. 3I; *n*=3/3).

### SHH-expressing cells make a small contribution to the ulna in a stage-dependent manner

**Fig. 4.**
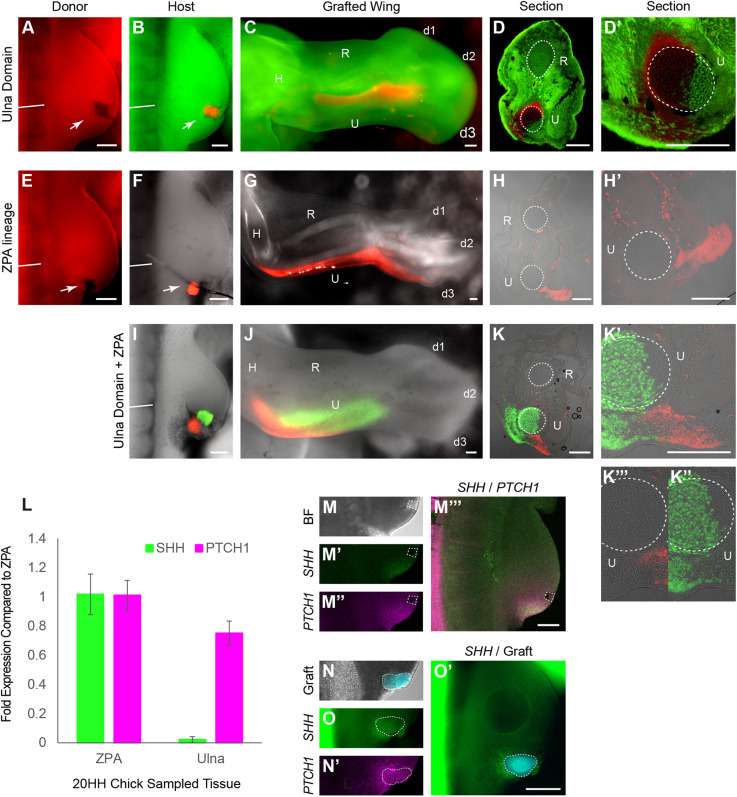
**ZPA lineage in chick wing in association with *SHH* and *PTCH1* expression.** (A,B) Confirmation of *Chameleon* results by homotopic grafting of presumed ulna from stage 20 HH tdTom chick wing bud (A) to eGFP chick wing bud (B). (C-D′) tdTom cells contribute to the entire length of the ulna (C) with sections confirming tdTom cells in cartilage (*n*=7/7) (D,D′). (E-H′) ZPA lineage determined through homotopic grafting of ZPA from stage 20 HH tdTom chick wing bud (E) to wild-type chick wing bud (F). tdTom cells do not contribute to the ulna (*n*=3) (as shown in wholemount, G; confirmed with sections, H,H′). White arrows indicate graft donor and host site. (I) Homotopic double grafts with ZPA derived from a tdTom chick wing bud and presumed ulna from an eGFP chick wing bud grafted into wild-type wing bud (*n*=3). (J-K‴) Subsequent wholemount (J) and sections (K-K‴) show that only eGFP cells contribute to ulna (*n*=3/3). Dashed white circles outline the ulna and radius in sections. (L) qRT-PCR for *SHH* (green) and *PTCH1* (magenta) performed for 20 HH ZPA, ulna and radius primordia. Data are mean±s.e.m. (M-M″) Close up of 20 HH chick wing with presumed ulna excised (dashed white box, shrinkage of excision site observed as an artefact of fixation) (*n*=3). HCR *in situ* hybridisation was then carried out for *SHH* (M′) and *PTCH1* (M″). (M‴) The same limb with merge of *SHH* and *PTCH1*. (N-O′) Close up of 20 HH chick wing with eGFP ZPA grafted into wild-type host (dashed white line) (N). HCR *in situ* hybridisation was then carried out for *SHH* (O) and *PTCH1* (N′) with merge of *SHH* and graft in O′. N,N′ and O,O′ are the same limb but imaged with confocal and fluorescent zoom microscopes, respectively (*n*=4). Straight white lines in A,B,E,F,I indicate the anterior-most edge of somite 19. H, humerus; R, radius; U, ulna; d1-d3, digits 1-3. Scale bars: 200 µm.

Our fate-mapping approach with *Chameleon*, like others before, creates small and discrete clones of labelled cells. Although excellent for generating fate maps with high spatial resolution, it does not demonstrate the fate of all the cells in a specific region. For example, no single bead application labelled all the cells of an ulna (Fig. 3D). To assess whether the area we had identified was able to generate all the cells of the ulna, we undertook homotopic grafting of the presumptive ulna primordia between stages 20 HH and 21 HH eGFP and tdTomato transgenic chicken embryos. Distal wing mesenchyme grafts from tdTomato stage 20 HH limbs, corresponding to the anterior of somite 19 and approximately 150×150 µm in size (Fig. 4A), were grafted into the equivalent area in eGFP embryos (Fig. 4B). Grafts of the presumptive ulna primordia (tdTom) gave rise to the cartilage of the ulna and carpals (*n*=7/7; Fig. 4C,D), and digit 3 (*n*=5/7) in host eGFP embryos. Unlike labelling of the ulna primordia via TAT-Cre application, contribution to the entire length of the ulna was dependent on graft size, as smaller grafts only gave rise to the distal ulna, carpals and digit (*n*=4/7). To confirm that grafts were correctly taken from the *SHH*^−^/*PTCH1*^+^ domain, we examined gene expression in donor limbs after grafts were excised, via HCR RNA *in situ* hybridisation, and in mock grafts, via qRT-PCR, which confirmed that the presumptive ulna primordia grafts were *SHH*^−^/*PTCH1*^+^ (Fig. 4M), confirming that the cells that generate the ulna at stage 20/21 HH come from within a distal *SHH*^−^ domain, an area outside the *SHH*-expressing ZPA, but still subject to SHH signalling.

It has previously been reported in mice that the ulna arises from *SHH*-expressing cells (Harfe et al., 2004), which conflicts with our result in chicken. Although in 3/7 stage 20/21 HH homochronic *SHH*^−^/*PTCH1*^+^ domain grafts we found that complete ulna developed from a SHH^−^ graft, we undertook further homotopic grafts of stage 20/21 HH chick ZPA cells (*SHH*^+^/*PTCH1*^+^) to ensure no contribution from the ZPA could be observed (Fig. 4E,F). RT-qPCR was used to confirm expression of *SHH* and *PTCH1* in ‘mock’ ZPA grafts when compared with ulna grafts (*P*<0.05, Fig. 4L) and HCR RNA *in situ* hybridisation confirmed grafted tissue that originated from the ZPA (*SHH*^+^/*PTCH1*^+^) was grafted into the ZPA region (*SHH*^+^/*PTCH1*^+^) in mock grafting experiments (*n*=4, Fig. 4N,O). All ZPA-ZPA stage 20 HH grafts contributed to the posterior mesenchyme of the limb at stage 33 HH but not the ulna (Fig. 4G,H). Finally, to explore the interaction between ZPA cells and the ulnar primordium, both the ZPA (tdTom) and the presumptive ulna region (eGFP) were transplanted together into wild-type stage 20 HH limb buds (Fig. 4I). At stage 33, there was no mixing of eGFP and tdTomato cells in all limbs (Fig. 4J). Only eGFP cells (*SHH*^−^/*PTCH1*^+^) were within the ulna cartilage, and ZPA-derived tdTom cells remained strictly outside the cartilage (Fig. 4K). These results demonstrate that at stage 20/21 HH, the chicken ulnar primordium is spatially defined outside the *SHH*-expressing ZPA, consistent with the original chicken limb fate maps of Saunders (1948) and Summerbell (1974), although it is subject to long-range SHH signalling.

**Fig. 5.**
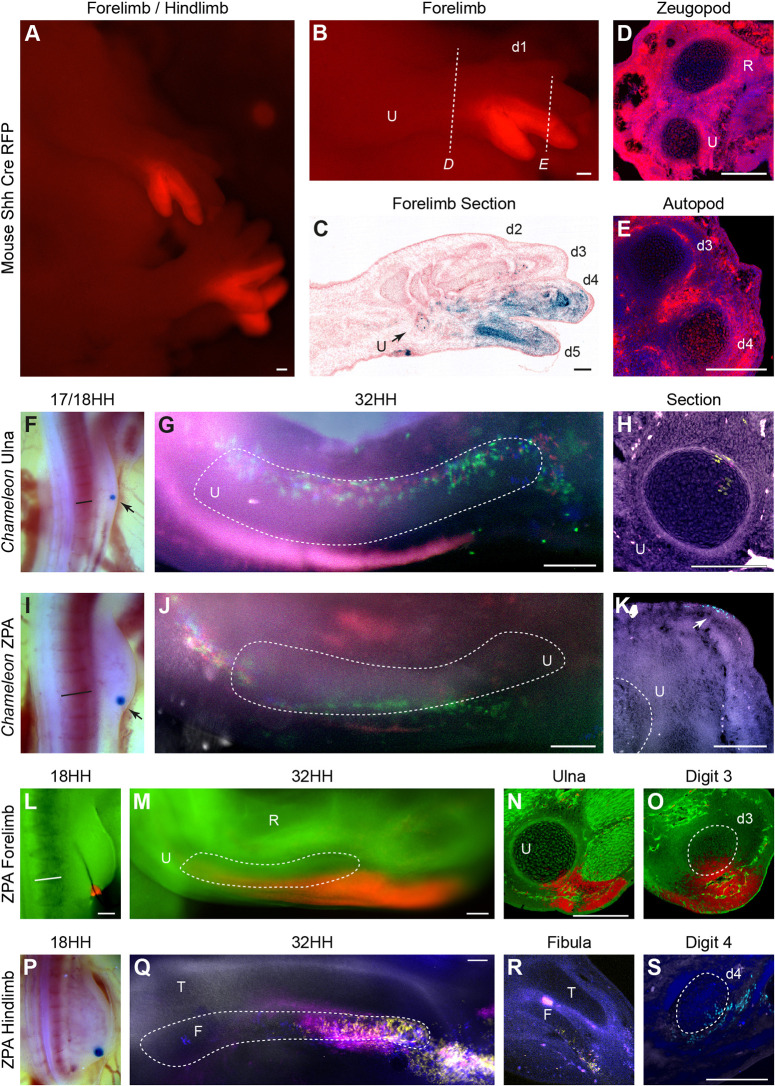
**ZPA lineage in forelimbs and hindlimbs of mice and 18 HH chicken.** (A) Right forelimb and hindlimb of an embryo carrying both Shh^tm1(EGFP/cre)Cjt^ and a Cre inducible tdRFP reporter with RFP expression highlighting cells in the *Shh* lineage (*n*=2 supplemented with publicly sourced databases and publications). (B) Same mouse with close up of forelimb with RFP in distal ulna and digits 4 and 5. (C) Longitudinal section from individual J:184579 (see Materials and Methods) with blue showing the *Shh* lineage. Black arrow indicates the distal ulna. Dashed lines in B indicate where sections of the zeugopod (D) and autopod (E) were taken with RFP in the cartilage of the ulna and digit 4. TAT-Cre bead placement (arrow) for the ulna in the *Chameleon* 17 HH wing bud (F) and for the ZPA in the *Chameleon* 18 HH wing bud (I), with subsequent wholemounts (G,J). (H,K) Sections show that fluorescent cells are within ulna cartilage for a bead placed outside the ZPA (*n*=5/5, H) but not for beads placed within the ZPA (*n*=4/4, K). Arrow in K indicates induced expression of Cytbow transgene in the ectoderm, an artifact of the experiment, but the ulna remains unlabelled. (L-O) Homotopic tdTom ZPA to eGFP grafts in 18 HH wing buds (L) to confirm contribution of tdTom to a minority of ulna cartilage but also to digit 3 cartilage (*n*=3/3) shown in wholemount (M) and sections (N,O). (P-S) TAT-Cre bead placement in the ZPA of *Chameleon* 18 HH hindlimb (P) with wholemount (Q) and sections (R,S) showing the ZPA lineage in the fibula and digit 4 (*n*=3). Straight black and white lines in F,I,L indicate the anterior-most edge of somite 19. HH, Hamburger Hamilton; R, radius; U, ulna; T, tibia; F, fibula; d1-d5, digits 1-5. Scale bars: 200 µm.

As the contribution of SHH ZPA cells to the ulna appears very different between our analysis in chicken and that published in mouse (Harfe et al., 2004; Scherz et al., 2007), we re-examined the Shh reporter mouse Shh^tm1(EGFP/cre)Cjt^ (mouse construction details in Materials and Methods). In combination with data from online resources (Baldarelli et al., 2021), we confirm that, although *Shh*-expressing cells do contribute to the ulna and posterior mesenchyme of the mouse forelimb zeugopod, localisation is primarily in the distal ulna and is far less extensive than the contribution to digits 4 and 5 (Harfe et al., 2004; Fig. 5A-E). Technical problems prevented us from investigating the contribution of *Shh*-expressing cells in the fibula of the Shh reporter mouse and although a section of the mouse fibula from online resources (Baldarelli et al., 2021) suggests there is a small contribution from Shh-expressing cells throughout the fibula, we are unable to comment on the total contribution of *Shh*-expressing cells in the mouse fibula.

Apical ectoderm ridge excision experiments of both Saunders (1948) and Summerbell (1974) suggest that the proximal chicken ulna begins specification before stage 20 HH, so we therefore sought to establish whether the difference between mouse and chicken data could be resolved by undertaking ZPA grafts earlier in development. We implanted TAT-Cre beads into the distal limb mesenchyme of stage 18 HH limbs at the axial level of anterior somite 19 (Fig. 5F) and more posteriorly into the ZPA (Fig. 5I). Localisation of fluorescent clones were substantially different between the experiments; beads placed in the ‘ulna region’ at anterior somite 19 resulted in fluorescent labelling in the stylopod, the cartilage of the ulna and a small contribution to the autopod (*n*=5/5; Fig. 5G,H). However, beads placed in the ZPA labelled the stylopod, posterior limb mesenchyme of the zeugopod and autopod, but not the ulna cartilage (*n*=4; Fig. 5J,K). We postulate that the stylopod contribution is due to the range of Cre-recombinase in the context of a smaller limb field relative to stage 20 HH limb buds. Therefore, we additionally undertook homotopic grafting of tdTom ZPA grafts to stage 18 HH eGFP embryos (Fig. 5L). In this instance, we found that tdTom ZPA grafts made a small contribution to posterior ulna and digit 3 (*n*=3/3; Fig. 5M-O), two of which also contributed to the full length of the ulna, confirming that, like the mouse, the chicken ulna has a small contribution of *SHH*-expressing cells but that the anatomical distribution of *Shh/SHH* is different between the species. *Shh*-expressing cells compose only the distal end of the mouse ulna but the entire proximo-distal length of the posterior chicken ulna.

The chick hindlimb foot has four digits and is considered to be a closer representative to the pentadactyl limb of mouse and human. The fourth digit of the chick leg is predominantly descended from ZPA cells, whereas the three anterior digits are not ZPA descendants, echoing ZPA contributions to digits in mice (Towers, 2018). The zeugopod of the chick hindlimb, fibula and tibia, are analogous to the ulna and radius of the forelimb, respectively. To examine whether the fibula, like the ulna of the mouse and chicken, also arises predominantly from *SHH*^−^/*PTCH1*^+^ cells, we implanted Tat-Cre beads into the ZPA of 18 HH hindlimb buds (Fig. 5P) and found, surprisingly, that the resulting fluorescent clones contributed to the distal two-thirds of the fibula, the fourth metacarpal and phalanges of digit 4 (Fig. 5Q-S). This suggests that a much larger contribution of *SHH*-expressing cells forms the fibula than forms the ulna. It also demonstrates that, even between the two posterior zeugopod bones of birds (i.e. ulna and fibula), there is a considerable difference in the cellular lineages that comprise them.

## DISCUSSION

Fate-mapping approaches have been fundamental in developmental biology and the chicken embryo has been particularly useful in developing anatomical and temporal fate maps of developing tissues due to its anatomical accessibility. Here, we demonstrate a new anatomical approach to fate-mapping that uses topically applied TAT-Cre to a transgenic chicken containing a Cre-inducible transgene. With the creation of stably labelled genetic clones in anatomically discrete areas, our approach faithfully recreates and improves on fate maps of the chicken limb made by Saunders (1948), Vargesson et al. (1997), Sato et al. (2007) and others, with cellular resolution amenable to sectioning, lightsheet microscopy and clonal analysis. We used our approach to comment on a long-held conundrum in limb development; the evolution of the tridactyl limb and homologies in the developing limbs of mice and chickens.

Evidence from the fossil record indicates that the path to the evolution of powered flight of modern birds from basal ground-based dinosaurs was likely multifactorial and piecemeal, requiring many anatomical changes in the skeletal, musculature, respiratory and integument systems (Brusatte, 2017; Brusatte et al., 2014; Dececchi and Larsson, 2009; Xu et al., 2014), including the loss of two digits (reviewed by Xu and Mackem, 2013). Fate mapping to establish the origin of avian digits, as well as ascertaining the contribution of SHH signalling and *SHH*-expressing cells to digits, has been used to support the evolutionary origin, and therefore digit identity, in modern birds (de Bakker et al., 2013; Kawahata et al., 2019; Tamura et al., 2011; Towers et al., 2011).

Conversely, the zeugopod element of the primary axis, i.e. the ulna, is treated as a fixed and unaltered point from which digit number and articulation subsequently change. There are only two bones in the zeugopod; thus, its post-axial position and earlier condensation in relation to the other bone, the radius, appear to satisfy the criteria for the ulna. This principle extends to the hindlimb, with the fibula recognised as being analogous to the ulna (Towers, 2018). Owing to the apparent conservation of the zeugopod skeleton, the distribution of ZPA descendants to the ulna of the pentadactyl limb in comparison with the tridactyl limb, or indeed with the fibula, has not yet been investigated.

**Fig. 6.**
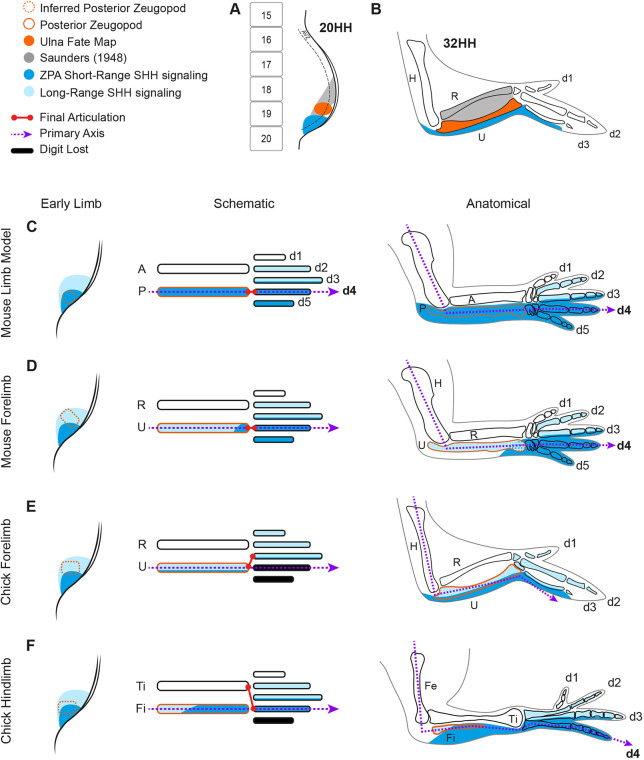
**ZPA lineage in forelimbs and hindlimbs of mice and 18 HH chicken.** (A,B) Updated fate map of the chick forelimb with the inclusion of our findings in solid orange. The ulna (B) arises from the anterior half of somite 19 in the distal 20 HH chick wing bud (A). (C-F) The posterior half of the early limb bud, schematic and anatomical representations of the mouse and chick limb bones. Dark blue represents the ZPA and its lineage; light blue depicts cells outside the ZPA that express direct SHH targets and their descendants, which are SHH dependent. Dotted orange lines demarcate the hypothetical area from which the posterior zeugopod originates. Dotted purple lines represent the primary axis, as defined by order of initial condensations. In the mouse fore- and hindlimbs (C,D), the primary axis goes through the posterior zeugopod and digit 4 with the ZPA contributing to digits 4 and 5, and to a posterior margin of digit 3. It is presumed that the posterior zeugopod arises from the ZPA (C; Harfe et al., 2004; Zhu et al., 2022). Current findings indicate that only the distal ulna of the mouse forelimb is derived from ZPA cells, demonstrating that the primary axis and ZPA lineage are not congruent (D). (E) ZPA cells contribute to the posterior-most ulna but, unlike the mouse, the entire length. If the primary axis is maintained through digit 4, then the chick wing has had no shift, maintaining the divergence of ZPA lineage to the primary axis. (F) Digit 4 and the majority of the fibula of the chick hindlimb are derived from ZPA cells, illustrating that the ZPA lineage of the posterior zeugopod bone is not conserved, even within species.

In the chicken, the ulna and fibula are dependent on SHH signalling, as shown by their absence in the OZD chicken (Ros et al., 2003), in which limb-specific SHH signalling is lost. We mapped the chick ulna in the stage 20 HH limb bud and showed that it consistently arises from a highly discrete area that is adjacent to anterior somite 19. This area is outside the ZPA but within the *PTCH1* expression domain, suggesting that the ulna is primarily subject to long-range paracrine SHH activity (Fig. 6A,B). However, forelimb ZPA grafts at an earlier stage (18 HH) give rise to the posterior margin of the ulnar cartilage. This implies that the ulna primordia domain moves from being partially within the ZPA, and subject to short-range SHH signalling, to an area wholly outside the ZPA while inside the window of zeugopod specification. Furthermore, we found that the majority of fibular cartilage is derived from *SHH*-expressing cells, indicative of being mostly subject to autocrine short-range SHH signalling. Even within the same species, the posterior zeugopod skeletal elements, the ulna and fibula, which are considered analogous to one another, have a large variation in the contribution of *SHH*-expressing cells. This is again different from the mouse ulna, which only has a small distal contribution of ZPA descendants. Although, like the chicken, the majority of the mouse ulna is likely subject to long-range paracrine SHH signalling, only the distal end of the ulna appears to be derived from cells subject to short-range autocrine Shh signalling.

Our finding that ZPA descendants contribute to the posterior margin of digit 3 cartilage in the chicken wing is different from both Towers et al. (2011), in which no ZPA descendants were found in the cartilage, and Tamura et al. (2011), in which digit 3 was entirely within the ZPA at 20 HH. We believe it is due to the greater enhancement in visualisation of grafted cells through the use of two transgenic reporter lines. However, digit 3 cartilage of the mouse also contains *Shh*-expressing cells and by this measure we interpret that the most posterior digit in the chicken wing is similar to digit 3 in the mouse, which does not change the interpretation of the axis-shift hypothesis. Digit 3 remains largely outside of the ZPA from as early as 18 HH, suggesting that a homeotic shift from digit 4 has not occurred in the autopod, as suggested by Tamura et al. (2011).

Digit identity, as determined by ZPA contribution, is often used as a principle for proposing hypotheses for limb variations across species but also interchangeably between the fore- and hindlimbs. However, we show that even structures considered to be fixed and conserved, including the posterior zeugopod bone, cannot be unified via the proportion of *SHH*-expressing cells in its makeup. This limitation has been acknowledged by others (Xu and Mackem, 2013) and RNA-sequencing of digits across five species, has shown that, apart from digit 1, there is little homology in the expression profiles over digits 2-5, including that of *SHH* (Stewart et al., 2019). We propose that the differences in ZPA contributions between the mouse ulna, chick ulna and chick fibula indicate the composite nature of a singular anatomical structure, consisting of a complicated underlying developmental course and a dynamic piecemeal evolutionary change of its own.

Our results also suggest that the primary axis and ZPA lineage are not consistently related (Fig. 6C-F). The ulna and fibula are acknowledged to be a fixed element of the primary axis, and digits have often been identified by their articulation and order of appearance in relation to the ulna or fibula (Larsson and Wagner, 2002; Shapiro et al., 2003). If there are variations in ZPA contributions between the ulna and fibula, and also between species, this suggests that the primary axis may also run through any digit regardless of its ZPA lineage, not only through digit 4, as has been the mainstay of digit identification. In mammals that demonstrate a reduction in SHH signalling and subsequently a loss of digits, such as the pig and cow, the primary axis is maintained via an articulation between ulna and digit 4 (Cooper et al., 2014; Tissieres et al., 2020). However, in birds with a delay and reduction in relative SHH signalling, which cause a loss of posterior digits and carpals, such as the emu, the ulna articulation shifts anteriorly to digit 3 (Kawahata et al., 2019; Smith et al., 2016), suggesting digit identity, as determined by SHH lineage, does not dictate the course of the primary axis.

In conclusion, we show the mouse and chicken ulna are predominantly not derived from *SHH*-expressing cells, suggesting they are subject to paracrine SHH signalling. Unlike the ulna, chick fibular cartilage is mostly descended from the ZPA and, thus, although the postaxial zeugopod is seen as fixed and often considered to be analogous, we demonstrate that these have different constituents of SHH lineage. The ulna and fibula may be more evolutionarily diverse than supposed and, therefore, their participation in the primary axis may be flexible and unrelated to ZPA lineage. We suggest that, with changes in digit number, the articulation of the zeugopod with the autopod have correspondingly developed to accommodate functionality over digit identity, and that the zeugopod will have adapted just as much as digits, alluded to by the variation in contributions of *SHH*-expressing cells.

## MATERIALS AND METHODS

### Chicken husbandry

ISA Brown, Roslin Green (Cytoplasmic GFP), Flamingo (TdTomato) and *Chameleon* (Cytbow) chicken lines were maintained under Home Office License at the Roslin Institute. Details of *Chameleon* line generation, and mapping of the insertion site can be found in Fig. S2. Fertilised chicken eggs were incubated at 38°C until the desired stage of embryonic development (Hamburger and Hamilton, 1951).

### Production of chameleon Tol2-Cytbow transgenic chicken line

A Tol2-Cytbow vector (a kind gift from Jean Livet, Xavier Morin and colleagues; Loulier et al., 2014; https://www.addgene.org/158991) and Tol2 transposase were transfected into Line Y2 and Line X5 wild type male primordial germ cells (PGCs; Novagen Brown chicken line) using C-DMRIE (Fig. S1A). After 12 days of culture (Whyte et al., 2015), cells were sorted with FACS for EBFP2. Positive cells were collected and expanded in FAOT media [B27 diluted DMEM supplemented with h-Activin (25 μg/ml), h-FGF2 (25 μg/ml) and ovotransferin (10 mg/ml)] at 37°C and 5% CO_2_ for several weeks, changing media every 3 days. After expansion, expression of EBFP2 was visualised (Fig. S1B). To test whether the transgene was functional and able to express tdTom, mCeru or mEYFP after Cre recombinase treatment, PGCs were differentiated as follows: between 0.5 μg-1 μg TAT-Cre recombinase protein was added to the culture medium and cells were examined for tdTom, mCeru or mEYFP protein expression (Fig. S1C). PGCs competent to express tdTom, mCeru or mEYFP were either frozen or injected directly into the dorsal aorta of 24 2-day-old chicken embryos (G_0_) as described by Macdonald et al. (2010) and Macdonald et al. (2012). Injected G_0_ chicken embryos were hatched in the National Avian Research Facility (NARF) (The Roslin Institute, Edinburgh, UK). These birds were raised to maturity and semen from male birds was screened by PCR for presence of the Tol2-Cytbow transgene. One male bird, CPX1-11, produced Tol2-Cytbow-positive semen and was subsequently bred to non-transgenic layer hens (ISA Brown). Fertile eggs from this cross were incubated to hatch to produce fully transgenic G_1_ birds. On hatching, positive G_1_ birds were identified by EBFP-positive chorioallantoic membrane tissue that had been collected from shells and examined for EBFP2 under a UV microscope. Positive male and female G_1_ birds were initially maintained at NARF and the line was named ‘*Chameleon’*; however, G_1_ females failed to produce eggs, a phenotype that was detrimental to their welfare, and they were subsequently culled. Thus, we have been unable to produce a homozygous *Chameleon* line and *Chameleon* is maintained as a heterozygous male line, which is crossed to non-transgenic layer hens (ISA Brown) to produce heterozygous *Chameleon* embryos. Mendelian ratios hold true in this cross as ∼50% of embryos produced in a *Chameleon*:ISA Brown cross contain the Tol2-Cytbow transgene, as screened by a handheld 365 nm black-light torch.

### PacBio sequencing

Day 6 whole embryos were screened for EBFP2 fluorescence, dissected from yolk and snap-frozen on dry ice in 1.5 ml microfuge tubes. Tubes were stored at −80°C until processed. Upon thawing, DNA was extracted using an NEB Monarch HMW DNA Extraction kit (T3060L) according to the manufacturer's protocol for frozen tissue. In total, eight embryos were extracted independently. Diluted genomic DNA was assessed using an Agilent TapeStation and quantified by a Qubit fluorometer. The best extraction, based on concentration and DNA integrity, was selected for library preparation. Library preparation and long-read sequencing were carried out by Edinburgh Genomics. The SMRTbell library was sequenced on a Pacific Biosystems Revio platform (25 M ZMW, HiFi mode) using a single SMRT cell. In total, 73 gigabases (Gb) were produced with an average fragment size of 14 kb and Phred quality of Q34. Data were returned in bam and fastq file formats.

### Transgene mapping

To detect the genomic location(s) of transgene insertion, hi-fi reads in fastq.gz format were first aligned against the corresponding transgene sequence (Addgene 158991) using Minimap2 (v2.24-r1122) (Li, 2021). In total, 131 out of 4,908,179 hi-fi reads were mapped. Transgene-mapped reads were retained by Samtools (v1.18 using htslib v1.18) (Danecek et al., 2021). The output file was converted from bam to fastq and the transgene-mapped reads were realigned using Minimap2 against the paternal haplotype of GRCg7w (white leghorn layer, GCF_016700215.2) (Smith et al., 2022). Of 131 reads, 97 were remapped to GRCg7w. Reads that failed to map were short and internal to the transgene; they were flagged as multimappers, presumably because of the homology shared between the fluorescent reporter genes. The remaining 97 reads mapped to two loci located at chromosomes 4 (NC_052576.1): 33493838-33512396 and 14 (NC_052586.1): 4149318-4179940. After further scrutiny, the latter locus defined by 27 uniquely mapped reads was determined to be an anomaly. The chromosome 14 locus corresponds to the location of the chicken β-actin gene (ACTB), the basal promoter sequence of which was used in the Tol2-Cytbow transgene construct to drive fluorophore gene expression. The chromosome 4 locus was defined by 53 uniquely mapped reads. Based on the transition of sequence homology from host genome to transgene, we defined the flanking coordinates of the Tol2-Cytbow transgene and piggyBac flanking insertion sites to reside between chromosome 4:33512395-33512402. The transgene insertion coordinates were defined and confirmed orthogonally by processing all hi-fi reads using Sniffles (v2.2) (Sedlazeck et al., 2018), a long-read structural variant caller. Crucially, no other transgene insertions were detected using Sniffles, thus confirming a single integration of the Tol2-Cytbow transgene on chromosome 4.

### Assessment of transgene expression after topical TAT-Cre recombinase application

To assess parameters of transgene recombination and tdTom/mCeru/mEYFP expression in response to topical TAT-Cre recombinase application, including the ratio of cells expressing tdTom/mCeru/mEYFP, the time from application of TAT-Cre Recombinase to detectable expression of tdTom/mCeru/mEYFP and duration of TAT-Cre Recombinase activity after application, experiments were initially undertaken *in vitro* in fibroblasts derived from PGCs (culture conditions above) and *ex ovo* in EC culture (Chapman et al., 2001; see supplementary Materials and Methods). Using parameters defined *ex ovo* to assess the range of TAT-Cre recombinase activity in limb bud mesenchyme, TAT-Cre recombinase-soaked beads (80-100 µM) were placed in stage 20 HH wing buds for 8 h (*n*=3). Embryos were fixed and cryosectioned (see below) then imaged on a Zeiss LSM880. Distance measurements were taken from the surface of the bead to all tdTom/mCeru/mEYFP using Zen Blue software, which was analysed in Microsoft Excel and plotted as a line graft in R. Using parameters defined *ex-ovo* to assess the duration of TAT-Cre recombinase activity, two beads were placed simultaneously in a limb bud and one was removed after 2 min (*n*=3). Comparison of labelling indicated that bead placement for 2 min (Fig. 1) produced similar results to those obtained if the bead was left in place, and these correspond to results found in *ex ovo* culture experiments (see supplementary Materials and Methods).

### Chameleon cytbow chicken manipulations

Fertilised eggs were windowed, staged (Hamburger and Hamilton, 1951) and checked for the transgene with a handheld 365 nM ‘black light’ torch (Alonefire 36 W 365 nM) and re-incubated until the appropriate stage. 80-100 µM Affi-gel Blue Gel beads (BioRad, 1537302) were prepared as described previously (Tiecke and Tickle, 2007) and placed in 1-2 µL TAT-Cre recombinase (EMD Millipore, SCR508, 1500 units) for a minimum of 30 min on ice. Beads were picked up on the tip of an electrolytically sharpened tungsten needle and allowed to shrink briefly, through air drying before insertion into the embryo. Embryos were manipulated directly from the incubator to maintain 38°C. Beads were left in place in the embryo unless otherwise stated. Eggs were then re-sealed with tape and incubated at 38°C in a humidified and light-free environment until the desired Hamburger and Hamilton stage. Embryos were culled in accordance with Schedule 1 of the Animals (Scientific Procedures) Act 1986. Embryos were dissected in ice-cold PBS, fluorescent expression visualised and assessed in wholemount on a Zeiss Axiozoom V16, using YFP, tdTom, CFP filters in preparation for further processing.

### Homotopic grafts

At the desired stage, host sites of Roslin Green or ISA Brown embryos were dissected and discarded using a tungsten dissecting needle. Donor sites from Flamingo embryos were dissected and moved into the host Roslin Green embryo via a p20 pipette containing DMEM. The graft was manoeuvred into the host site and, when necessary, secured with a piece of 0.02 mm oxidised nickel chrome wire. Care was taken to ensure ectoderm orientation was maintained between donor and host. Embryos for whole-mount analysis were culled and dissected at around stage 33 HH, fixed and cleared with CUBIC reagent 1 before being imaged on a Zeiss Axiozoom V16 microscope. Embryos for HCR *in situ* hybridisation were allowed to incubate for 3 h after graft insertion, then culled and dissected in ice-cold DEPC PBS before being fixed with 4% PFA at 4°C overnight.

### Mouse construction and genotyping

Mice used in this study were housed at the animal facilities at the University of Edinburgh, with procedures performed under Personal and Project Home Office Licences. Male mice carrying the Shh^tm1(EGFP/cre)Cjt^ allele (Harfe et al., 2004) were mated to female mice carrying a Cre reporter line (Luche et al., 2007). Cre expression leads to excision of a floxed transcriptional Stop cassette and allows expression of the tdRFP in all descendant cells. Embryos were collected at E14.5, genotyped by standard methods and fixed overnight in 4% PFA. Publicly sourced images of mouse J:184579 can be obtained The Jackson Laboratory (Baldarelli et al., 2021; https://www.informatics.jax.org/recombinase/specificity?id=MGI:3053959&system=respiratory+system and https://images.jax.org/webclient/img_detail/17489).

### Hybridisation chain reaction *in situ* hybridisation

Whole-mount tissue was prepared for HCR by dissecting in ice-cold DEPC PBS and fixing in 4% PFA overnight. After washing twice in PBT for 5 min each, fixed tissue were dehydrated with a series of methanol/PBST washes for 5 min each on ice. Once dehydrated in up to 100% methanol, tissue were stored in −20°C until further use. Before performing HCR, tissues were rehydrated with a series of methanol/PBST washes for 5 min on ice up to 100% PBST. Tissues were treated with 10 μg/ml proteinase K solution at room temperature for a length of time that was calculated at 15 s per stage (e.g. 5 min for stage 20 HH). These were post-fixed in 4% PFA at room temperature, then washed twice in PBST for 5 min each, 50% PBST/50% 5×SSCT for 5 min, then 5×SSCT for 5 min, all on ice. We then performed HCR v3.0 using the protocol as described by Molecular Instruments (Choi et al., 2018). Split initiator probes (v3.0) for PTCH1 (accession NM_204960.2) and SHH (accession NM_204821.1) were designed by Molecular Instruments.

### Sections

Embryos were dissected in ice-cold PBS and fixed in 4% PFA overnight. After sucrose treatment, limbs were embedded in a solution of 7.5% gelatin and 15% sucrose in PBS then frozen in isopentane at around −60°C and stored at −80°C until sectioning. Serial sections were obtained with a Bright OTF5000 cryostat microtome at a 10 μm and mounted on Polysine Adhesion microscope slides. Once dry, slides were washed in PBS at 37°C and mounted with coverslips. Images were obtained on the LSM880 confocal microscope using Zen Black software.

### Tissue clearing

Tissue clearing was used to improve visualisation of deep fluorescent clones in whole-mount limb tissue. Fixed tissues were washed in PBS for 5 min at room temperature then submerged in CUBIC reagent 1A (as per Susaki et al., 2015) at 37°C for 2-6 h until cleared and imaged on a Zeiss Axiozoom V16.

### RT-qPCR

Five samples of ulna and ZPA were dissected from 20 HH ISA Brown embryos and batched for a single reaction. These were stored at −80°C before RNA extraction using Pre cellys bead homogenisation (Bertin Technologies, France) and RNA easy Kit (Qiagen). Turbo DNA free DNase kit (Ambion) was used to remove genomic DNA contamination before cDNA was synthesised using AffinityScript Multiple Temperature cDNA Synthesis Kit (Agilent) using Oligo DT. Triplicate qRT-PCR reactions were carried out per biological replicate using an MX 3005P thermal cycler (Agilent) with a FAST 2 step thermal cycling protocol (95°C for 10 s, 60°C for 30 s). Brilliant iii Ultra Fast SYBR green qPCR master mix (Agilent) and chicken primers were used at 100 nM final concentration and were as follows: LBR F, GAAGCTGCAGTACCGGATCA; LBR R, GCTAGGTCTTCCTCAGGTGC (housekeeping gene); SHH (accession #NM_ 204821.1) F, CCAAATTACAACCCTGAC; SHH R, CATTCAGCTTGTCCTTGCAG; PTCHD1 F, TGGGAAATACAATTCCACCTTC; PTCHD1 R, CTCCAGGAGGACAACATTTCA. Data were analysed using MX Pro software and exporting to Excel where a 2-ddCT method was used to calculate relative expression compared with ZPA.

## Supplementary Material



10.1242/develop.202340_sup1 Supplementary information
