# Supplementary material for: Insights into digit evolution from a fate map study of the forearm using Chameleon, a new transgenic chicken line
Source: Development. 2024 Jun 28;151(13):dev202340. doi: 10.1242/dev.202340 (PMC11234372; doi:10.1242/dev.202340)
Supplement: Supplementary information [file develop-151-202340-s1.pdf]

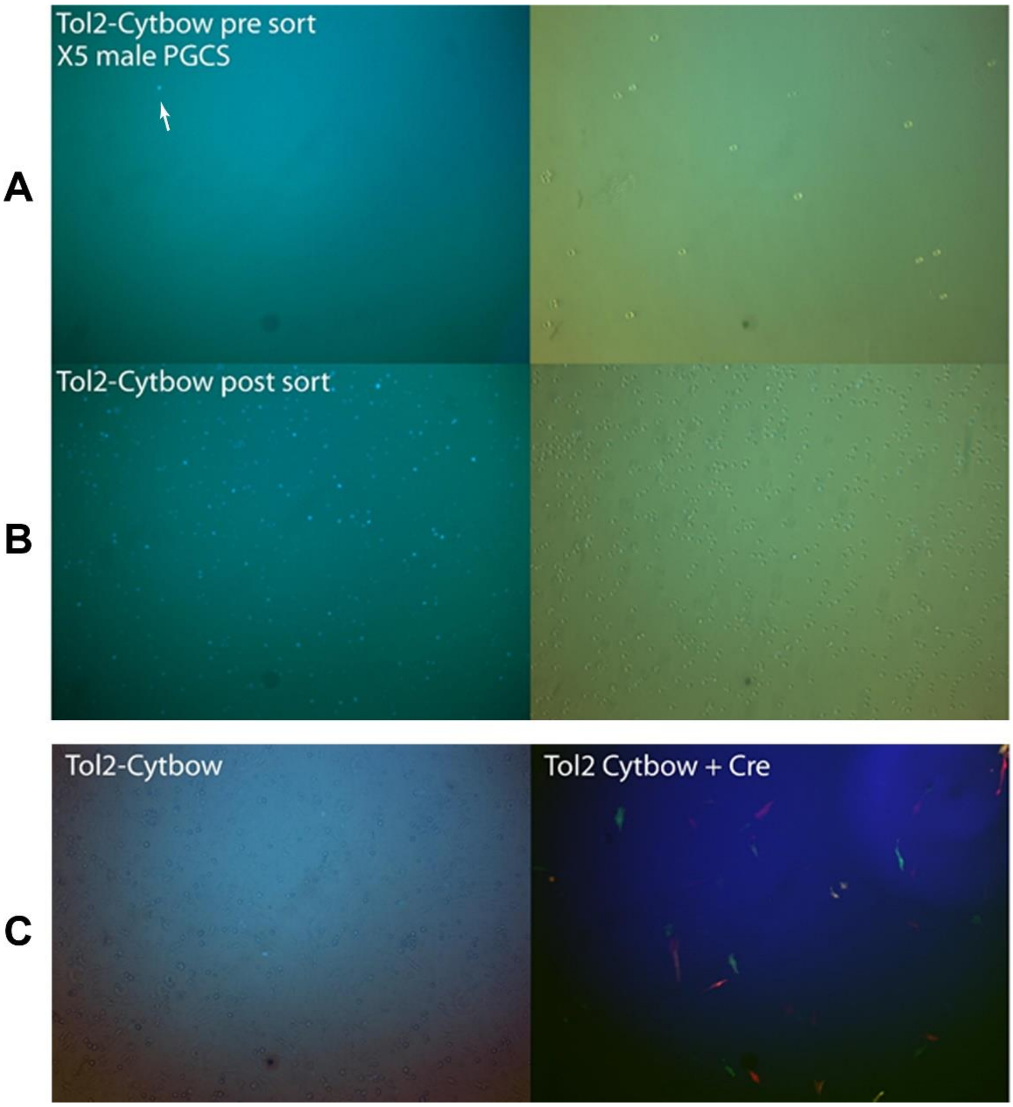

Fig. S1. Expression of Tol2-Cytbow in transfected PGCs

LineX5 wild type male PGCs after transfection of Tol2-Cytbow. **(A)** Transfected PGCs are sparse as observed under transmitted light (right panel) and show infrequent expression of EBFP2 (arrow left panel) as observed with a EBFP2 filter. **(B)** Transfected PGCs after successful expansion and FACS for EBFP2 positive cells, showing a higher concentration of EBFP2 positive cells as observed under transmitted light (right panel) and with a EBFP2 filter (left panel). **(C)** Adherent cells, differentiated from EBFP2+ PGCs, expressing tdTom, mCeru or mEYFP (right panel) after addition of Cre Recombinase to the cell culture medium. Not all cells express tdTom/mCeru/mEYFP and some cells retain EBFP2 expression.

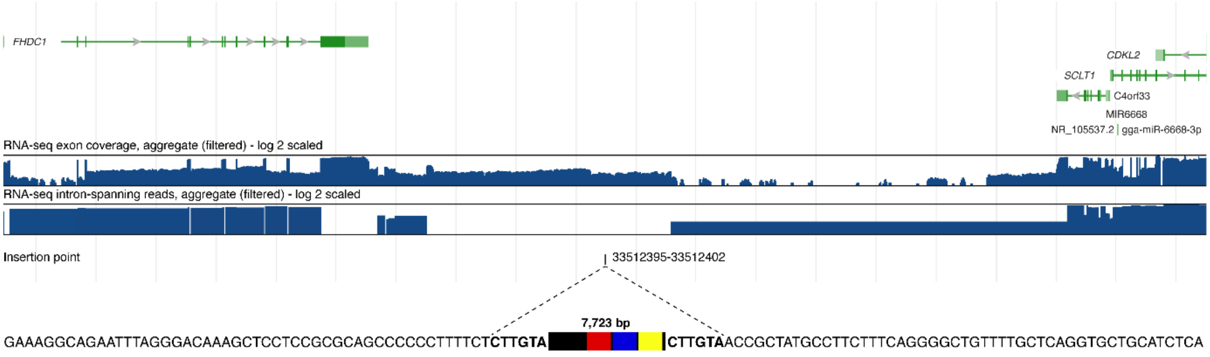

Fig. S2. Mapped location of Tol2-Cytbow in *Chameleon*

CPX transgene insertion at chromosome 4 locus. The illustration is adapted from the National Library of Medicine's Genome Data Viewer at coordinates chr4:33.465-33.555 Mb locus (bGalGal1.pat.whiteleghornlayer.GRCg7w, NCBI Gallus gallus annotation release 106). The insertion point is enlarged to depict flanking host sequence, duplicate insertion site (boldface sequence), and transgene (coloured block). The transgene is not depicted to scale with respect to flanking sequence.

Development • Supplementary information

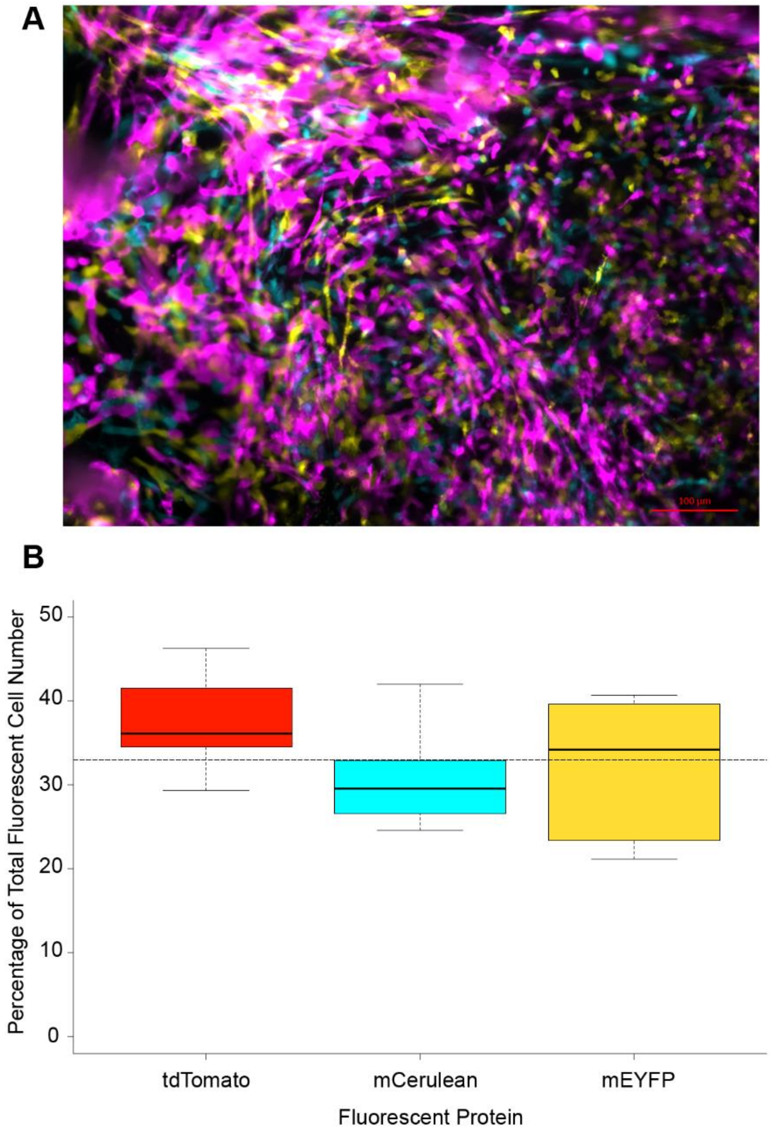

Fig. S3. ***Chameleon*** **cells expressed tdTomato, mCerulean or mEYFP in equal ratio after TAT-Cre Recombinase application**

(A) Culture of adherent *Chameleon* cells differentiated from PGCs 9 hours after 0.75µL of TAT-Cre Recombinase was pipetted into the culture media. The induced fluorophores, tdTomato, mCerulean or mEYFP, can all be observed and no cell expresses more than one colour. Scale bar = 200µm. **(B)** Boxplot showing percentage of each fluorescent colour seen in the total number fluorescent cells (n=10). The dashed line indicates 33.33% of the total percentage of fluorescent cells (α=0.01. dtTom: µ=37%, p=0.0414. mCeru: µ=30%, p=0.1356. mEYP2 µ=32%, p=0.7186.)

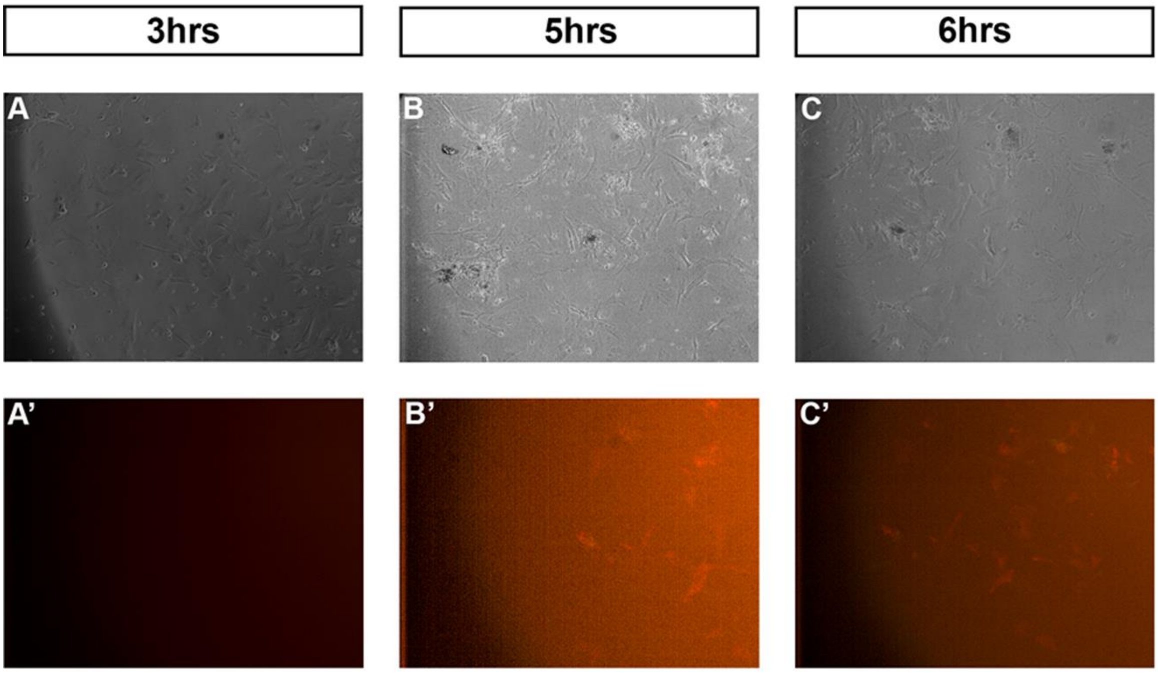

Fig. S4. Expression of tdTomato is observed in vitro 5 hours after TAT-Cre Recombinase application in *Chameleon* cells via a Zeiss Live Cell Observer

Fluorescence is first detected at around 5 hours after the addition of 0.5µl of TAT-Cre to adherent cultures of CPX primordial germ cell. The fluorescent signal grows stronger over the course of the next several hours. Top panels are transmitted light showing all cells. Bottom panels showing tdTom positive cells at 3 hours (A, A'), 5 hours (B, B') and 6 hours (C, C').

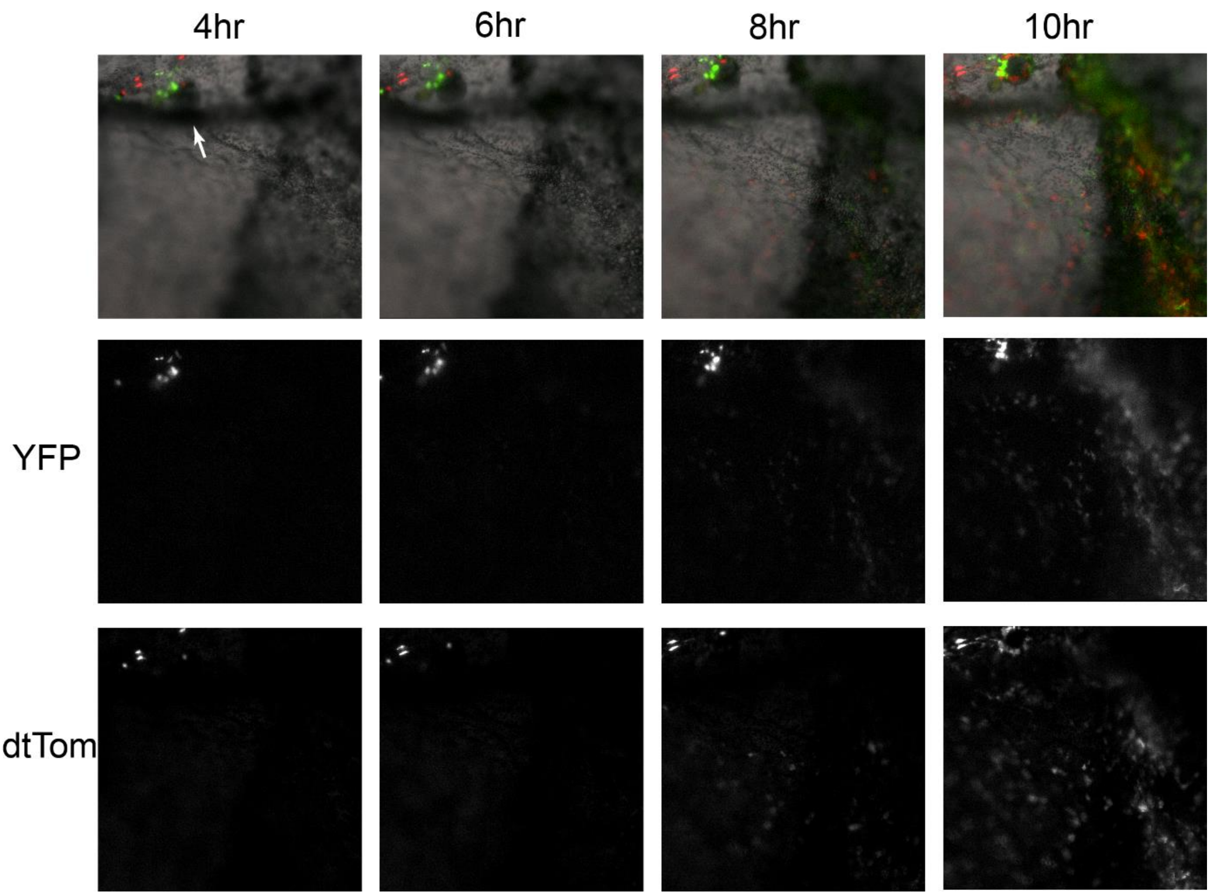

Fig. S5. Expression of tdTomato is observed 6 hours after TAT-Cre Recombinase application in *Chameleon* EC Culture using a Nikon Eclipse Ti microscope

Ex ovo EC culture Chameleon embryo imaged on a Nikon Eclipse Ti with mercury bulb illumination source, on which it was possible to observe only tdTom and mEYFP. A bead soaked in TAT-Cre had previously been applied to the embryo (arrow) to induce tdTom and mEYFP in order to allow the correct image capture settings (i.e. exposure) to capture expression of newly induced tdTom and mEYFP which is observed widely to the right of the image. Weak tdTom and mEYFP can be observed at 6 hours, which become stronger over time.

Development • Supplementary information

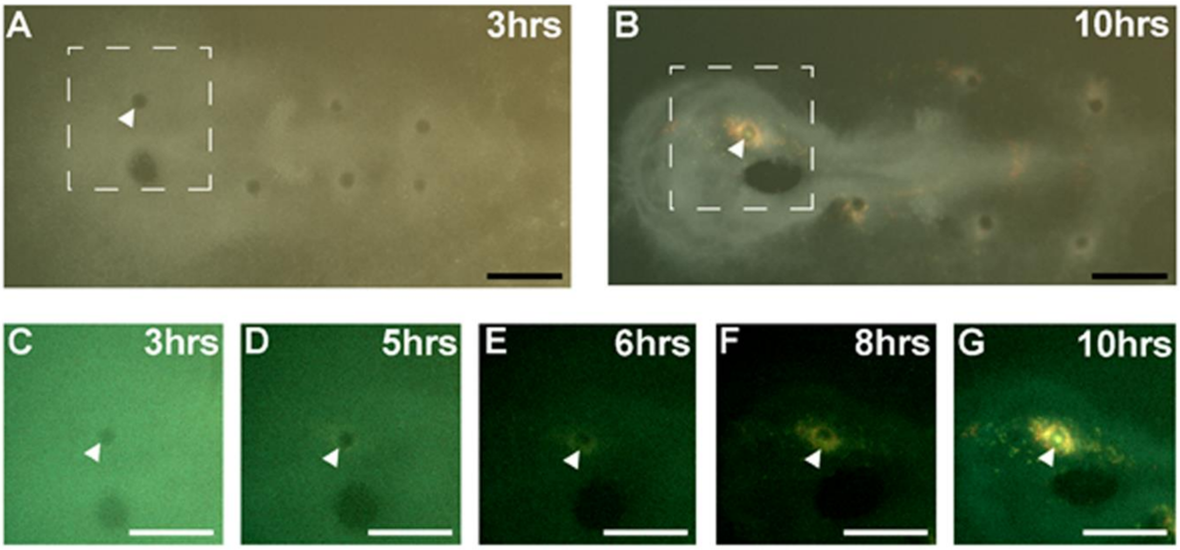

Fig. S6. Expression of tdTom/mCeru/mEYFP is observed 5 hours after TAT-Cre Recombinase application in *Chameleon* EC Culture using Zeiss Axiozoom V16 microscope

Ex ovo EC culture *Chameleon* embryo imaged on a Zeiss Axiozoom V16 (HXP 200C LED excitation), on which it is possible to observe tdTom/mCeru/mEYFP2 expression. **(A)** In a *Chameleon* embryo 3 hours after six TAT-Cre soaked Affi-Gel Blue beads have been placed along the antero-posterior axis (anterior to the left), no tdTom/mCeru/mEYFP expression can be observed. **(B)** The same embryo after 10 hours, in which it in now possible to observe tdTom/mCeru/mEYFP expression around all six beads. **(C-F)** Magnification of area in **(A)** outlined by a white box, showing bead indicated by white arrowhead in **(A)**, showing no expression of tdTom/mCeru/mEYFP at 3 hours **(C)** but weak expression at 5 hours **(D)**, increasing over 6 hours **(E)** and 8 hours **(F)** until strong expression is seen at 10 hours (**G**; magnification of area within white box in **B**). All scale bars = 200µm.

Development • Supplementary information

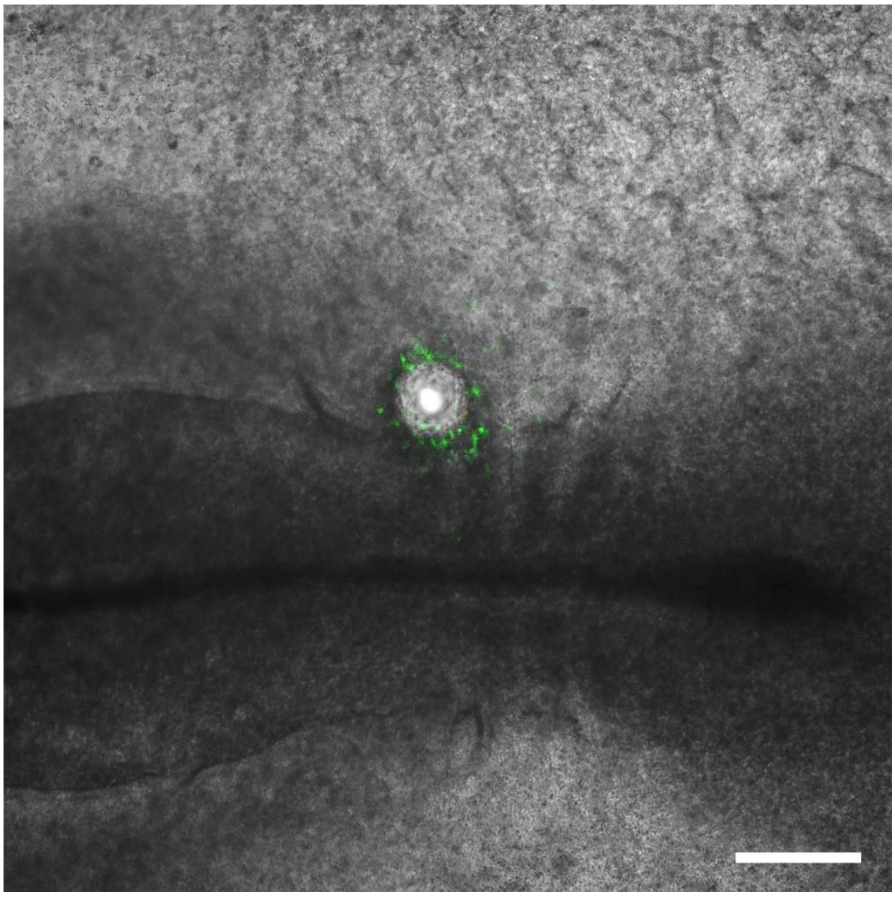

Fig. S7. Expression of tdTom/mCeru/mEYFP is observed 2 hours after TAT-Cre Recombinase application in *Chameleon* EC Culture using Zeiss LSM880 microscope

Ex ovo EC culture *Chameleon* embryo imaged at 2 hours after bead application on a Zeiss LSM880 confocal microscope, showing expression of induced mEYFP expression around the bead. Scale bar = 200µm

Development • Supplementary information

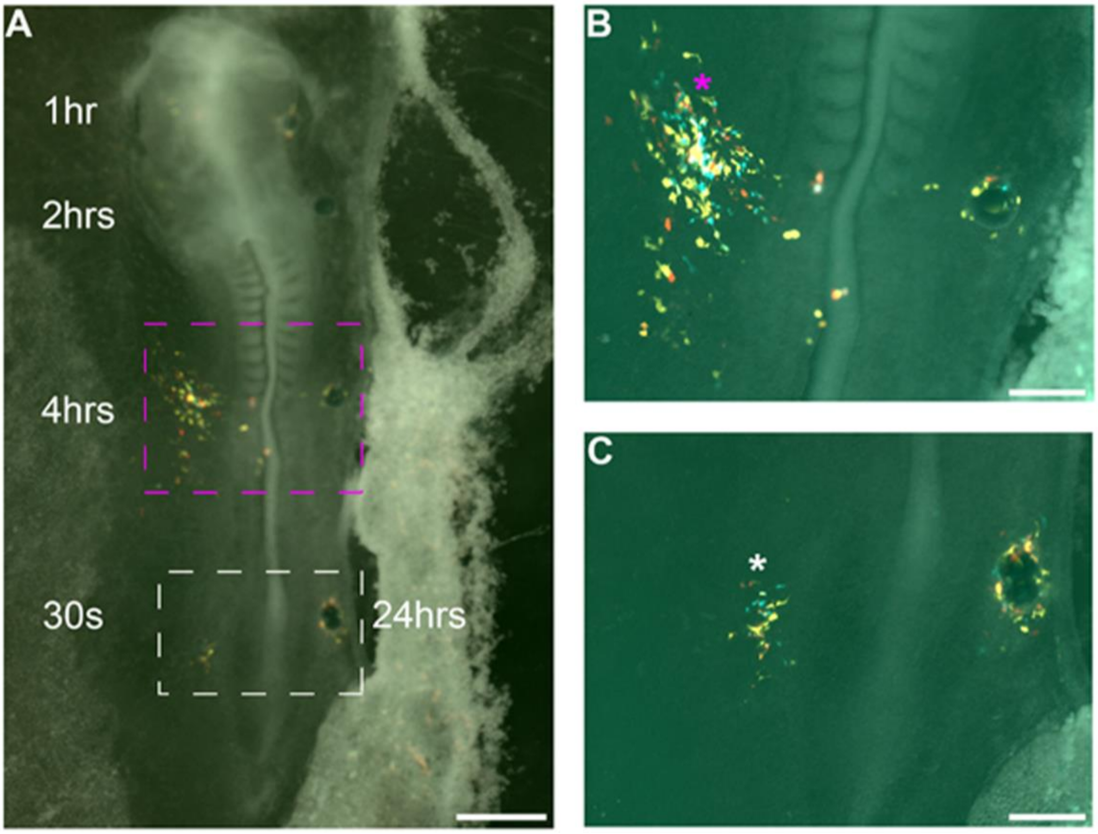

Fig. S8. The action of topical TAT-Cre Recombinase is immediate and transient when applied via Affi-Gel Blue Beads in ex ovo *Chameleon* EC culture

A 30 second application of a TAT-Cre bead induces fluorescence in *Chameleon* embryo EC culture. **(A)** Stage 10HH *Chameleon* embryo after 24 hour incubation with TAT-Cre beads. The location of the beads removed from the left side after 30 seconds, 1 hour, 2 hours and 4 hourss is indicated and fluorescence can be seen in these areas. The four contralateral beads can be seen on the right side of the embryo, surrounded by fluorescence (n=4). **(B)** The discrete populations of fluorescent cells induced by the bead removed after 4hrs (magenta asterisk) and by the bead opposite that had been left for 24 hours. The cells labelled by the 24 hour bead are a much smaller and more localised population than those labelled by the 4 hour bead. **(C)** The discrete populations of fluorescent cells induced by the bead removed after 30 seconds (white asterisk) and by the bead opposite that had been left for 24 hours. The labelled populations are similar in localisation and size. Magenta box: the area shown in **(B)**. White box: the area shown in **(C)**. Magenta asterisk: the cells labelled by the 4 hours bead. White asterisk: the cells labelled by the 30 second bead. Scale bars = 500µm **(A)**; 200µm **(B, C)**.

Supplementary Materials and Methods

Determining the ratio of expression of tdTom/mCeru/mEYFP and time to visualisation after application of TAT-Cre Recombinase application in vitro

Primordial germ cells were extracted from the blood of *Chameleon* line embryos and cultured in 250µl of FAOT media at 37ºC and 5% CO_2 for several weeks, changing media every 3 days. To make adherent cells were transferred to a well coated in gelatin and fed with 500µl chicken growth media (CGM; 10% FCS, 1% chick serum and penicillin/streptomycin, 50µl ovalbumin). Cells were incubated for 8 days until 100% confluence of adherent cells. Then adherent PGCs were trypsinised and 100µl of cells were plated onto glass chamber slides in 400µl of CGM. The chamber slides were incubated for three days until adherent cells could be seen in the wells. Different wells received different volumes of TAT-Cre pipetted straight onto the adherent cells: 0.75, 0.5, 0.35, and 0.25µl. Cells were imaged 24 hours after TAT-Cre Recombinase application via a Zeiss Axiozoom V16 microscope (Fig. S3A). Images of fluorescent *Chameleon* PGCs were opened in FIJI/ImageJ (Schindelin et al., 2012), and converted to an 8-bit image. Each colour channel was thresholded separately using the default thresholding method, to account for differences in exposure/brightness. The 'analyse particles' function was used to automatically quantify the number of cells (size: 50-infinity pixels2; circularity: 0-1). Data was recorded in excel, saved as comma delimited files (.csv) and imported into R or RStudio. Base R graphics were used to visualise the box and whisker plots (Fig. S3B). To assess the time at which expression of the tdTom fluorophore could be visualised, adherent *Chameleon* cells were incubated on a Zeiss Cell Observer at 38ºC and 5% CO_2.

Determinning the time to visualisation of tdTom/mCeru/mEYFP after application of TAT-Cre Recombinase in ex ovo EC culture

To examine the dynamics of expression of tdTom/mCeru/mEYFP after addition of TAT-Cre Recombinase in an embryo, we utilised the Early Chick (EC) culture protocol from Chapman et al. (2001). 6 well culture plates or 35mm culture plates were prepared with 2ml of EC culture medium (1:1 thin albumin (from unfertilised eggs): agar (0.179% NaCl, 0.6% Bacto-Agar (Difco) in dH2O)). Embryos were collected with a filter paper mount, by placing a filter paper with a hole in the centre over the embryo and attaching to the vitelline membrane. Yolk

Development • Supplementary information

and other remnants are cleaned by placing the embryo in Ringer's solution or PBS and embryos were cultured ventral side up on the solidified culture medium. Embryos were incubated on either a Nikon Eclipse Ti (mercury bulb UV excitation; Fig. S5), Zeiss Axiozoom V16 (HXP 200C LED excitation; Fig. S6) or Zeiss LSM880 (confocal laser; Fig. S7) at 37ºC, with images taken of embryos between every 10-30 minutes, depending on imaging set-up.

Determining the duration of topically applied TAT-Cre Recombinase activity via Affi-Gel Blue Beads in ex ovo EC culture

To examine the duration that TAT-Cre Recombinase is active if applied topically via an Affi-Gel Blue bead, we undertook the following experiments *ex ovo* in EC culture (as described above). In EC *ex ovo* culture, eight TAT-Cre Recombinase soaked beads were placed in anterior to posterior sequence on endoderm, either side of the midline. Beads on the right side were left in place for the duration of the experiment, while beads on the left side were removed after 30 seconds, 1 hour, 2 hours and 4 hours and visualised on a Zeiss AxioZoom V16 microscope after 24 hours of incubation.

Supplementary References

Chapman, S. C., Collignon, J., Schoenwolf, G. C.and Lumsden, A. (2001). Improved method for chick whole-embryo culture using a filter paper carrier. *Dev Dyn.* **220,** 284-9.

Schindelin, J., Arganda-Carreras, I., Frise, E., Kaynig, V., Longair, M., Pietzsch, T., Preibisch, S., Rueden, C., Saalfeld, S., Schmid, B., et al. (2012). Fiji: an open-source platform for biological-image analysis. *Nat Methods.* **9,** 676-82.

Development • Supplementary information
