# Supplementary material for: Insights into digit evolution from a fate map study of the forearm using Chameleon, a new transgenic chicken line
Source: Development. 2024 Jun 28;151(13):dev202340. doi: 10.1242/dev.202340 (PMC11234372; doi:10.1242/dev.202340)
Supplement: Supplementary file 1 [file develop-151-202340_review_history.pdf]

Insights into digit evolution from a fate map study of the forearm using *Chameleon*, a new transgenic chicken line

Julia Dong Hwa Oh, Lu Freem, Dillan David Zachary Saunders, Lynn McTeir, Hazel Gilhooley, Melany Jackson, James D Glover, Jonathan Smith, Jeffrey J Schoenebeck, Laura A Lettice, Helen M Sang and Megan G Davey
DOI: 10.1242/dev.202340

Editor: James Briscoe

Review timeline
Original submission: 8 September 2023
Editorial decision: 27 October 2023
First revision received: 21 February 2024
Editorial decision: 18 March 2024
Second revision received: 17 May 2024
Accepted: 20 May 2024

Original submission

First decision letter

MS ID#: DEVELOP/2023/202340

MS TITLE: Insights into Digit Evolution from a Fate Map Study of the Forearm

AUTHORS: Julia Dong Hwa Oh, Dillan David Zachary Saunders, Lynn McTeir, Melany Jackson, James D Glover, Jeffrey J Schoenebeck, Laura A Lettice, and Megan G Davey

I have now received all the referees' reports on the above manuscript, and have reached a decision. The referees' comments are appended below, or you can access them online: please go to BenchPress and click on the 'Manuscripts with Decisions' queue in the Author Area.

As you will see, the referees express considerable interest in your work, but have some significant criticisms and recommend a substantial revision of your manuscript before we can consider publication. All the referees agree that the application of the new chick Cytbow line is an important feature of this study. As such, it would be important to include more details of the methods - duration, diffusion, and other parameters of the Cre delivery, etc. The study would also be strengthened if the observation of the difference in contribution of the ZPA lineage to the long bones of the wing and leg was followed up experimentally and the implications discussed. The referees also provide several suggestions for clarifications and corrections.

If you are able to revise the manuscript along the lines suggested, which may involve further experiments, I will be happy to receive a revised version of the manuscript. Your revised paper will be re-reviewed by one or more of the original referees, and acceptance of your manuscript will depend on your addressing satisfactorily the reviewers' major concerns. Please also note that Development will normally permit only one round of major revision. If it would be helpful, you are welcome to contact us to discuss your revision in greater detail. Please send us a point-by-point response indicating your plans for addressing the referees’ comments, and we will look over this and provide further guidance.

© 2024. Published by The Company of Biologists under the terms of the Creative Commons Attribution License (https://creativecommons.org/licenses/by/4.0/).

Please attend to all of the reviewers' comments and ensure that you clearly highlight all changes made in the revised manuscript. Please avoid using 'Tracked changes' in Word files as these are lost in PDF conversion. I should be grateful if you would also provide a point-by-point response detailing how you have dealt with the points raised by the reviewers in the 'Response to Reviewers' box. If you do not agree with any of their criticisms or suggestions please explain clearly why this is so.

Reviewer 1

Advance summary and potential significance to field

This paper describes a pioneer study employing the cytbow chicken transgenic model (in addition to other transgenic chicken resources) to fate map the zeugopod bone progenitors, with particular reference to whether they are under the influence of autocrine or paracrine Shh signaling. The significance of the results in contributing to the debates on digit number assignment, the primary limb axis and digit reduction in avians are discussed.

The results make a contribution to contemporary debates on digit assignment and digit evolution. The transgenic chicken experimental approach used has several technical advantages over methods employed in the past.

Comments for the author

Comments on the results:

Fig.2D there do seem to be a few labelled cells just posterior to the radius. This would not impact any of the conclusions made by the authors but could this be commented on?

It is interesting that the Shhcre mouse reagent labels a (perhaps) surprisingly, distally-restricted population compared to the chick ZPA grafts, which leave a trail of cells along the proximal-distal extent of both zeugopod and autopod.

Perhaps related, Figure 5 chick schematic-coloured marks extend to distal extreme but perhaps should not as graft method used most likely would have left some cells (unlabelled) just under the AER and these would have contributed to distal most phalanx/phalanges.

Could the forelimb and hindlimb difference be due to heterochrony between the forelimb and hindlimbs?

Minor typographical
Line 92 “in the a foundation principal,
Line 116 SHH-expressings ZPA cells
193 represented by small clones of labelled cells….. fate napping

272 J:184579 unclear what this refers to

Line 301 The chick hindlimb comprises of four digits

Cpx terminology in Figure 4 not defined in main text that I could find.

Line 413 “we show although the mouse and chicken ulna are predominantly SHH-,” this could be expressed more clearly.

Reviewer 2

Advance summary and potential significance to field

In this paper the authors want to investigate the cellular and genetic contribution to ulna development mainly in the avian wing, as a means to understand the evolution of the avian primary

© 2024. Published by The Company of Biologists under the terms of the Creative Commons Attribution License (https://creativecommons.org/licenses/by/4.0/).

limb axis and digits that articulate with it. With this aim they use a novel genetic fate mapping technique they have developed in chicks, which is complemented by classical grafting experiments involving distinct fluorescent reporter chicken lines. By employing these approaches, the authors establish that most of the ulna develops from a region anterior to the ZPA (Shh-Ptch1+) and they also observe that ZPA descendants also contribute to the very posterior ulna, carpals and digit 3 in the chick. The study clearly shows that the contribution of the ZPA to the ulna in chick has previously been overlooked due to temporal factors. Additionally, they also show that in the leg a bigger proportion of the fibula derives from ZPA cells. The authors discuss the differences noted in the contribution of ZPA descendants, both between the ulna and fibula in the chick and between the chick and mouse ulna and the implications for the primary axis.

Comments for the author

The experiments are well-conceived, and the results very nicely presented. I would like to highlight several points that I believe can contribute to the improve the paper.

First, regarding the use of the Chameleon cytbow chicken with the TAT-Cre recombinase locally applied in beads. Although there is a reference to an accompanying paper describing the method, I recommend including here the relevant information about the duration, diffusion, and other parameters of the Cre delivery from beads.

Major points:

- First, with regard to the use of the Chameleon cytbow chicken with TAT-Cre recombinase applied locally in beads, while there is a reference to an accompanying paper detailing the method, I would recommend including in this paper the pertinent information concerning the dynamics (duration, diffusion, and other parameters) of the Cre delivery from the bead.

- The Introduction includes several imprecise statements. For instance, in line 32, the authors assert that the HH20 mesoderm is already fated, although it might be more accurately to describe it as being specified.
The statement in lines 50-54 is also imprecise, as it remains unclear whether the observed phenotypes in the AER-Fgfs mutants are a result of altered proliferation. Furthermore, the statement regarding the absence of an ulna in the Shh-deficient limbs in different species (lines 58-60) is inaccurate. Indeed, only applies to the chick ozd mutant. In humans, the element distal to the humerus is notably dysplastic and cannot be definitively identified as either ulna or radius. A similar situation is observed in mice, where only one element forms in the forelimb zeugopod, devoid of distinct identity. Additionally, recent research has revealed that the digit that persists in mice, while conventionally labeled as digit 1, originates from the ZPA (Zhu et al. 2022).

- Given the comparison between the chick ulna and fibula, I believe that an analysis of the ZPA's contribution to the fibula in the mouse is required to provide a comprehensive analysis. Such an analysis would introduce a novel aspect, as opposed to the well-documented forelimb analysis.

- I suggest a more thorough integration and discussion of the authors' results. For instance, the HCR demonstrate that the anterior boundary of the Ptch1 expression domain extends further anterior than that of Shh, indicating long-range signaling. It would be valuable to discuss these findings in the context of recent observations in the mouse (Zhu et al. 2022).

- The authors consider their results support Towers' conclusions, and they nicely discuss the flexibility of the primary axis. However, I miss some discussion on whether their results rule out the alternative homeotic transformation model.

- While acknowledging that I am not an expert in evolution, I express my reservations regarding the recurring connection between the reduction of digit number and powered flight.

-

Minor points:

© 2024. Published by The Company of Biologists under the terms of the Creative Commons Attribution License (https://creativecommons.org/licenses/by/4.0/).

- I recommend incorporating the supplementary figures into the main manuscript
- Please clarify mouse construction?
- please specify the number of samples analyzed for each experiment

Reviewer 3

Advance summary and potential significance to field

This paper highlights a new method for very precise lineage analysis in chick.
Results clearly demonstrate the utility of direct TAT-Cre application in ovo used in conjunction with a chick line transgenic for conditional fluorescent reporters. The technique is novel and has the potential for much greater precision in lineage tracing than achieved with either focal dye labeling in the chick model, or using tissue-specific Cre drivers in mouse.

Comments for the author

In this manuscript, Oh et al. use a clever strategy for precise lineage tracing to re-examine the Shh-producing ZPA lineage in chick limb bud, focusing on contribution of ZPA descendants to the posterior zeugopod elements (ulna fibula). The ZPA lineage has been of interest as one criterion for comparing digit identity across species, particularly in the context of digit reduction because ZPA descendants give rise to digits 4-5 and has been used as evidence that in chick wing digits 4-5 are lost (no ZPA-descendant digits form). But the zeugopod lineage has been less carefully analyzed. Here the authors use direct TAT-Cre protein application to embryos in ovo to activate conditional floxed fluorescent reporters from a chameleon transgenic chick line (with cytbow reporters). Since TAT-Cre enters cells directly, recombination is limited to cells immediately adjacent to a loaded bead.

The technique is novel and has potential for much greater precision in lineage analysis than achieved with either focal dye labeling in the chick model, or using tissue-specific Cre drivers in mouse. Overall, the experiments are well executed and demonstrate the utility of the transgenic reporter/TAT-cre protein system. However, the results of the lineage analysis are largely confirmatory of past work in chick and in mouse.

The one result that is new and unexpected is the striking difference in extent of Shh-descendant contribution to posterior zeugopod in forelimb vs hindlimb (ulna vs fibula), which isn't discussed at all. This difference is unique to chick and not seen in the mouse (Fig. 4A), and points to a difference in the extent of the Shh/ZPA in chick hindlimb compared to mammal even though the chick hindlimb is viewed as being more basal/ancestral than wing. Although the morphologic changes in chick fibula don't become apparent until a much later stage, might this somehow be related to differences between the mammalian and avian fibula (which is far more reduced)? Could this be checked by reducing Shh activity level in early hindlimb bud?

The appeal of the paper could be broadened with more focus and detail on the novel TAT-Cre methodology used (the results are a clear proof of principle).
This technique was previously used to examine lineage in chick skin explants (Ho et al, 2019), but whereas the utility of TAT-Cre in cultures has been clear for decades, its use in situ in embryos has not been evaluated as far as I can tell (the reference cited, IJDB 2018, appears to be a perspective/review where TAT-Cre use is mentioned without giving much detail). The authors indicate that a paper has also been submitted to Bio-protocols; I don't know if that precludes highlighting the technique here as well.

Specific comments on the experiments and text follow below. The paper is also very long, and could be shortened.

1. Some background discussion is oversimplified:
In the introduction (line 58) and elsewhere, the authors refer to “posterior” digit loss in the mouse Shh-KO based on morphology. But by criteria of ZPA lineage, the single remaining digit in the Shh-

© 2024. Published by The Company of Biologists under the terms of the Creative Commons Attribution License (https://creativecommons.org/licenses/by/4.0/).

KO is in fact a posterior (digit 4-5) ZPA-derived descendant, albeit with very transformed morphology (see Zhu 2022).

In several places, ZPA derived digits are described as being patterned in response to autocrine Shh signaling. The extent of this contribution is still a matter of debate. Autocrine response, at least in the mouse, becomes quite limited over time, with much of the ZPA being non-responsive (see Ahn and Joyner, 2004; Zhu, 2022).

2. In Fig. 3 – the excised graft defect in the donor limb in Fig. 3A looks much larger than that in Fig. 3M''' (same scale) where the defect location relative to Shh and Ptch1 expression is analyzed. This is relevant because the authors make the argument that the donor graft encompasses a Shh-responding but not Shh-expressing zone. Is the size difference an artifact of distortion from fixation/HCR protocols? Or is there variability in the graft size used?

3. In Fig 4 – why there is stylopod labeling after TAT-Cre application to the ZPA region in very early stage limb bud is not clearly explained in the text.
There's no evidence that Shh descendants contribute to stylopod, either in chick or mouse. Presumably this is because of exposure of adjacent cells destined to contribute to future stylopod in the context of a very small limb bud with proximal and distal zones are in very close proximity.

4. In Fig. 5 and legend, it's not clear what is meant by "presumed" mouse lineage and why the particular references chosen are cited in regard to this?
The Zhu, 2022 reference focuses only on hindlimb, and doesn't discuss ZPA lineage representation in the zeugopod at all. The Krawchuk, 2010 paper implies that Shh regulates posterior zeugopod specification but doesn't suggest any lineage relationship. The Harfe, 2004 paper showed that ZPA descendants contribute to ulna and could be viewed as assuming the contribution to ulna to be broad; wouldn't this be the more appropriate ref. to cite?

First revision

Author response to reviewers' comments

Summary of changes to the original submission

All reviewers requested more details on the generation and use of the *Chameleon* chicken. We have now added a full description of the generation of the *Chameleon* line, from transfection of primordial germ cells through to generation of the F0 and F1 birds, genome sequencing and mapping of the location of the transgene. This is in the Supplementary Information, as although it is important information regarding the *Chameleon* line, it is not directly relevant to the paper. We have also included in the main body of the paper a description of two important parameters applicable to use of this chicken line when TAT-Cre recombinase is applied topically in the limb bud, specifically the distance from the source of TAT-Cre recombinase that recombination and subsequent fluorescent proteins expression occurs, when TAT- Cre recombinase is applied on a Affi-gel Blue Gel bead, and the duration that topically applied TAT-Cre recombinase continues to be active. This forms a new figure (Figure 2). We complement this data with further Supplementary data on the equal expression of the induced fluorophores, the time of expression of induced fluorophores after application of TAT-Cre recombinase, the distance of expression of induced fluorophores from localised application of TAT-Cre recombinase via a Affi-gel Blue bead in ex-ovo EC culture and the duration of TAT-Cre recombinase action via analysis of expression of induced fluorophores in ex-ovo EC culture. Again, we consider this to be important information regarding the use of the *Chameleon* line but it is not directly relevant to limb development, and it is lengthy, so we have not included it in the main body of the paper. This work was undertaken by several post-docs and post-graduate students who have now been added as authors to the paper.

We thank the reviewers for their time and responses and feel we have addressed all outstanding points possible, which, we think, has made the paper more succinct and correct.

© 2024. Published by The Company of Biologists under the terms of the Creative Commons Attribution License (https://creativecommons.org/licenses/by/4.0/).

Megan Davey and Julia Oh

Detailed replies to the Reviewers

Reviewer 1 Advance Summary and Potential Significance to Field...

This paper describes a pioneer study employing the cytbow chicken transgenic model (in addition to other transgenic chicken resources) to fate map the zeugopod bone progenitors, with particular reference to whether they are under the influence of autocrine or paracrine Shh signaling. The significance of the results in contributing to the debates on digit number assignment, the primary limb axis and digit reduction in avians are discussed.

The results make a contribution to contemporary debates on digit assignment and digit evolution. The transgenic chicken experimental approach used has several technical advantages over methods employed in the past.
Many thanks for your comments. We have included a new results section to include the generation of the Chameleon line and the parameters for the induction of fluorescence in the presence of Cre-recombinase.

Reviewer 1 Comments for the Author...

Comments on the results:

Fig.2D there do seem to be a few labelled cells just posterior to the radius. This would not impact any of the conclusions made by the authors but could this be commented on?
We have added a remark to address this point in the figure legends (now Figure 3; Experimental artefact (arrowhead)) and have included details concerning labelling in the new results section as well as in the supplementary data.

It is interesting that the Shhcre mouse reagent labels a (perhaps) surprisingly, distally-restricted population compared to the chick ZPA grafts, which leave a trail of cells along the proximal-distal extent of both zeugopod and autopod.
We show these differences in Figure 6 (was Figure 5; summary figure) and discussion (Line 325-327) but have added further comment in the results (Line 251-252).

Perhaps related, Figure 5 chick schematic-coloured marks extend to distal extreme but perhaps should not as graft method used most likely would have left some cells (unlabelled) just under the AER and these would have contributed to distal most phalanx/phalanges.
Figure 6 (was Figure 5) is not solely a summary of our grafts but a composite of all the information thus far including Tower et al., 2011 work where there is contribution of the ZPA to the very distal end, albeit not cartilage, of the autopod.

Could the forelimb and hindlimb difference be due to heterochrony between the forelimb and hindlimbs?
We cannot address the heterochrony between forelimbs and hindlimbs in this paper but in the same stage chick, the hindlimb is more advanced than the forelimb so we would have expected less labelling with the ZPA grafts.

Minor typographical

Line 92 “in the a foundation principal,
Changed to ‘embodied in a foundation principle’ (Line 108)
Line 116 SHH-expressings ZPA cells
Changed to ‘*SHH* expressing ZPA cells’ (Line 134)
193 represented by small clones of labelled cells….. fate napping
This part of the results section has been removed.

272 J:184579 unclear what this refers to

© 2024. Published by The Company of Biologists under the terms of the Creative Commons Attribution License (https://creativecommons.org/licenses/by/4.0/).

MGI Ref. ID – we have added more information for clarification (Baldarelli et al., 2021)
https://www.informatics.jax.org/recombinase/specificity?id=MGI:3053959&system=respiratory+system#refSection

Line 301 The chick hindlimb comprises of four digits
Changed to 'The chick hindlimb foot has four digits' (Line 272)

Cpx terminology in Figure 4 not defined in main text that I could find.
Changed to Chameleon

Line 413 "we show although the mouse and chicken ulna are predominantly SHH-," this could be
expressed more clearly.
Changed to 'we show the mouse and chicken ulna are predominantly not derived from SHH expressing cells' (Line 362)

Reviewer 2 Advance Summary and Potential Significance to Field...

In this paper the authors want to investigate the cellular and genetic contribution to ulna development mainly in the avian wing, as a means to understand the evolution of the avian primary limb axis and digits that articulate with it. With this aim they use a novel genetic fate mapping technique they have developed in chicks, which is complemented by classical grafting experiments involving distinct fluorescent reporter chicken lines. By employing these approaches, the authors establish that most of the ulna develops from a region anterior to the ZPA (Shh-Ptch1+) and they also observe that ZPA descendants also contribute to the very posterior ulna, carpals and digit 3 in the chick. The study clearly shows that the contribution of the ZPA to the ulna in chick has previously been overlooked due to temporal factors. Additionally, they also show that in the leg a bigger proportion of the fibula derives from ZPA cells. The authors discuss the differences noted in the contribution of ZPA descendants, both between the ulna and fibula in the chick and between the chick and mouse ulna and the implications for the primary axis.

Reviewer 2 Comments for the Author...

The experiments are well-conceived, and the results very nicely presented. I would like to highlight several points that I believe can contribute to the improve the paper.

First, regarding the use of the Chameleon cytbow chicken with the TAT-Cre recombinase locally applied in beads. Although there is a reference to an accompanying paper describing the method, I recommend including here the relevant information about the duration, diffusion, and other parameters of the Cre delivery from beads.

Major points:

First, with regard to the use of the Chameleon cytbow chicken with TAT-Cre recombinase applied locally in beads, while there is a reference to an accompanying paper detailing the method, I would recommend including in this paper the pertinent information concerning the dynamics (duration, diffusion, and other parameters) of the Cre delivery from the bead.
Many thanks for your kind words. We have included a new results section with additional supplementary data to include the generation of the Chameleon line and the parameters for the induction of fluorescence in the presence of Cre-recombinase.

The Introduction includes several imprecise statements. For instance, in line 32, the authors assert that the HH20 mesoderm is already fated, although it might be more accurately to describe it as being specified.

© 2024. Published by The Company of Biologists under the terms of the Creative Commons Attribution License (https://creativecommons.org/licenses/by/4.0/).

Changed to 'specified' (Line 47)

The statement in lines 50-54 is also imprecise, as it remains unclear whether the observed phenotypes in the AER-Fgfs mutants are a result of altered proliferation.
These lines have been deleted as it was not necessary to discuss the effects of FGFs in this paper.

Furthermore, the statement regarding the absence of an ulna in the Shh-deficient limbs in different species (lines 58-60) is inaccurate. Indeed, only applies to the chick ozd mutant. In humans, the element distal to the humerus is notably dysplastic and cannot be definitively identified as either ulna or radius. A similar situation is observed in mice, where only one element forms in the forelimb zeugopod, devoid of distinct identity. Additionally, recent research has revealed that the digit that persists in mice, while conventionally labeled as digit 1, originates from the ZPA (Zhu et al. 2022).
We have modified this statement to address these differences. (Line 68 -76)

Given the comparison between the chick ulna and fibula, I believe that an analysis of the ZPA's contribution to the fibula in the mouse is required to provide a comprehensive analysis. Such an analysis would introduce a novel aspect, as opposed to the well-documented forelimb analysis.
We have attempted to analyse the mouse fibula and to increase the numbers of the mouse but have been unable to retrieve any tissue. Limited data from a publicly available database (https://www.informatics.jax.org/recombinase/specificity?id=MGI:3053959&system=respiratory+system#refSection) would suggest that SHH cell contribution is sparse and limited to the distal end of the fibula.
Although the forelimb digits have been well-documented, we would argue that the forelimb zeugopod has been far less described. Towers et al., 2011 and Tamura et al., 2011 do not discuss the chicken ulna in the context of ZPA lineage. Scherz et al., 2007 do not discuss the ulna in relation to SHH exposure. Harfe et al., 2004 do comment that approximately one-third of the mouse ulna is derived from the ZPA but do not feature the ulna in the model of temporal gradient of Shh exposure. Zhu et al., 2022 make an observation of anatomy which contributes to a model of SHH cell contribution in their paper based on SHH signaling manipulation but do not show the full equivalent data for the forelimb compared to the autopod.

I suggest a more thorough integration and discussion of the authors' results. For instance, the HCR demonstrate that the anterior boundary of the Ptch1 expression domain extends further anterior than that of Shh, indicating long-range signaling. It would be valuable to discuss these findings in the context of recent observations in the mouse (Zhu et al. 2022).
We have added a section in the Discussion figure (now Figure 6) to demonstrate the *PTCH1* and *SHH* boundaries and how the presumed ulna fits in this context.

The authors consider their results support Towers' conclusions, and they nicely discuss the flexibility of the primary axis. However, I miss some discussion on whether their results rule out the alternative homeotic transformation model.
A comment on the frameshift model has been added to the discussion (Line 335-337).

While acknowledging that I am not an expert in evolution, I express my reservations regarding the recurring connection between the reduction of digit number and powered flight.
We have deleted most references to powered flight.

Minor points:

I recommend incorporating the supplementary figures into the main manuscript
We have implemented this suggestion and have added supplementary Figure 1 to the new Figure 3 and supplementary Figure 2 to the new Figure 4

Please clarify mouse construction?
Mouse construction details are in the Materials and Methods and we have added a signpost for this in the results section (Line 151)

please specify the number of samples analyzed for each experiment

© 2024. Published by The Company of Biologists under the terms of the Creative Commons Attribution License (https://creativecommons.org/licenses/by/4.0/).

We have included additional sample numbers in the results section and more specifically, in the figure legends as per manuscript guidelines.

Reviewer 3 Advance Summary and Potential Significance to Field...

This paper highlights a new method for very precise lineage analysis in chick.
Results clearly demonstrate the utility of direct TAT-Cre application in ovo, used in conjunction with a chick line transgenic for conditional fluorescent reporters. The technique is novel and has the potential for much greater precision in lineage tracing than achieved with either focal dye labeling in the chick model, or using tissue-specific Cre drivers in mouse.

Reviewer 3 Comments for the Author...

In this manuscript, Oh et al. use a clever strategy for precise lineage tracing to re-examine the Shh-producing ZPA lineage in chick limb bud, focusing on contribution of ZPA descendants to the posterior zeugopod elements (ulna, fibula). The ZPA lineage has been of interest as one criterion for comparing digit identity across species, particularly in the context of digit reduction, because ZPA descendants give rise to digits 4-5 and has been used as evidence that in chick wing digits 4-5 are lost (no ZPA- descendant digits form). But the zeugopod lineage has been less carefully analyzed. Here the authors use direct TAT-Cre protein application to embryos in ovo to activate conditional floxed fluorescent reporters from a chameleon transgenic chick line (with cytbow reporters). Since TAT-Cre enters cells directly, recombination is limited to cells immediately adjacent to a loaded bead.

The technique is novel and has potential for much greater precision in lineage analysis than achieved with either focal dye labeling in the chick model, or using tissue-specific Cre drivers in mouse. Overall, the experiments are well executed and demonstrate the utility of the transgenic reporter/TAT-cre protein system. However, the results of the lineage analysis are largely confirmatory of past work in chick and in mouse.
Many thanks for your kind comments. We have included a new results section with additional supplementary data to include the generation of the Chameleon line and the parameters for the induction of fluorescence in the presence of Cre-recombinase.

Although the forelimb digits have been well-documented, we would argue that the forelimb zeugopod has been far less described. Towers et al., 2011 and Tamura et al., 2011 do not discuss the chicken ulna in the context of ZPA lineage. Scherz et al., 2007 do not discuss the ulna in relation to SHH exposure. Harfe et al., 2004 do comment that approximately one-third of the mouse ulna is derived from the ZPA but do not feature the ulna in the model of temporal gradient of Shh exposure. Zhu et al., 2022 make an observation of anatomy which contributes to a model of SHH cell contribution in their paper based on
SHH signaling manipulation but do not show the full equivalent data for the forelimb compared to the autopod.

The one result that is new and unexpected is the striking difference in extent of Shh-descendant contribution to posterior zeugopod in forelimb vs hindlimb (ulna vs) This difference is unique to chick and not seen in the mouse (Fig. 4A), and points to a difference in the extent of the Shh/ZPA in chick hindlimb compared to mammal even though the chick hindlimb is viewed as being more basal/ancestral than wing. Although the morphologic changes in chick fibula don't become apparent until a much later stage, might this somehow be related to differences between the mammalian and avian fibula (which is far more reduced)? Could this be checked by reducing Shh activity level in early hindlimb bud?
One of the main discoveries of this paper which we have discussed and which forms the basis of Figure 6 (was Figure 5) is the difference of the contribution of SHH to forelimb and hindlimb. This study cannot comment on whether this forms the basis for the reduction of the avian fibula which forms later during development. In the discussion, we comment on the SHH OZD chicken mutant which has lost a fibula.

© 2024. Published by The Company of Biologists under the terms of the Creative Commons Attribution License (https://creativecommons.org/licenses/by/4.0/).

The appeal of the paper could be broadened with more focus and detail on the novel TAT-Cre methodology used (the results are a clear proof of principle). This technique was previously used to examine lineage in chick skin explants (Ho et al, 2019), but whereas the utility of TAT-Cre in cultures has been clear for decades, its use in situ in embryos has not been evaluated as far as I can tell (the reference cited, IJDB 2018, appears to be a perspective/review where TAT-Cre use is mentioned without giving much detail). The authors indicate that a paper has also been submitted to Bio-protocols; I don't know if that precludes highlighting the technique here as well.

Specific comments on the experiments and text follow below. The paper is also very long, and could be shortened.

1. Some background discussion is oversimplified: In the introduction (line 58) and elsewhere, the authors refer to "posterior" digit loss in the mouse Shh-KO based on morphology. But by criteria of ZPA lineage, the single remaining digit in the Shh-KO is in fact a posterior (digit 4-5) ZPA-derived descendant, albeit with very transformed morphology (see Zhu, 2022).
We have clarified this point to say 'loss of posterior digit identity' (Line 69)

In several places, ZPA derived digits are described as being patterned in response to autocrine Shh signaling. The extent of this contribution is still a matter of debate. Autocrine response, at least in the mouse, becomes quite limited over time, with much of the ZPA being non-responsive (see Ahn and Joyner, 2004; Zhu, 2022).
Throughout the paper, we have described the ZPA derived cells are subject to autocrine SHH signaling. As pointed by the referee, the work in the mouse by Ahn Joyner/Zhu describe that the SHH derived cells become unresponsive over time and growth is instrumental after a burst of SHH signaling. However, in the chicken, paracrine and autocrine signaling still pattern limb throughout the period which we study. Although we believe the patterning mechanisms are conserved between mouse and chicken, the data is not yet aligned. Our efforts here we think align the fundamental patterning of the autopod more than has achieved before and hope that it continues to the ongoing debate. We have also added long-range vs short-range to the paper.

2. In Fig. 3 – the excised graft defect in the donor limb in Fig. 3A looks much larger than that in Fig. 3M''' (same scale) where the defect location relative to Shh and Ptch1 expression is analyzed. This is relevant because the authors make the argument that the donor graft encompasses a Shh-responding but not Shh-expressing zone. Is the size difference an artifact of distortion from fixation/HCR protocols? Or is there variability in the graft size used?
This is an artefact of fixation. We have added a comment in the Figure 4 (was Figure 3) legend.

3. In Fig 4 – why there is stylopod labeling after TAT-Cre application to the ZPA region in very early stage limb bud is not clearly explained in the text. There's no evidence that Shh descendants contribute to stylopod, either in chick or mouse. Presumably this is because of exposure of adjacent cells destined to contribute to future stylopod in the context of a very small limb bud with proximal and distal zones are in very close proximity.
The latter is our understanding of why there are fluorescent cells in the stylopod. The beads used in 17/18HH are the same size as ones used in 20HH and in the context of an even smaller limb bud, cells proximal/anterior to the bead at 20HH will likely contribute to the stylopod. To mitigate these technical issues, a more accurate representation of the contribution of the ZPA was shown via the 18HH ZPA grafts. We have added the following to the results:
'We postulate that the stylopod contribution is due to the range of Cre-recombinase in the context of a smaller limb field relative to stage 20HH limb buds. Therefore, we additionally undertook homotopic grafting of dtTom ZPA grafts to stage 18HH eGFP embryos (Fig. 5L). In this instance we found that dtTom ZPA grafts made a small contribution to posterior ulna and digit 3 (n=3/3; Fig. 5M-O), two of which also contributed to the full length of the ulna..' (Line 263-268)

4. In Fig. 5 and legend, it's not clear what is meant by "presumed" mouse lineage and why the particular references chosen are cited in regard to this? The Zhu, 2022 reference focuses only on hindlimb, and doesn't discuss ZPA lineage representation in the zeugopod at all. The Krawchuk, 2010 paper implies that Shh regulates posterior zeugopod specification but doesn't suggest any lineage relationship. The Harfe, 2004 paper showed that ZPA descendants contribute to ulna and could be viewed as assuming the contribution to ulna to be broad; wouldn't this be the more appropriate ref. to cite?

© 2024. Published by The Company of Biologists under the terms of the Creative Commons Attribution License (https://creativecommons.org/licenses/by/4.0/).

The posterior zeugopod in the summary figure of Zhu, 2022, whether it represents the ulna or fibula in a cartoon that appears to be a forelimb, is designated as a structure that is specified directly by SHH similar to digits 4 and 5. This is what we have represented in the 'presumed mouse limb'. We have omitted Krawchuk et al 2010 and added Harfe et al (2004).
Our interpretation of the figures in Zhu, 2022 is that the hindlimb fibula derives from GLI1 progenitors but not SHH progenitors, suggesting paracrine, indirect specification similar to digits 2 and 3.

Second decision letter

MS ID#: DEVELOP/2023/202340

MS TITLE: Insights into Digit Evolution from a Fate Map Study of the Forearm Using 'Chameleon', a New Transgenic Chicken Line

AUTHORS: Julia Dong Hwa Oh, Lu Freem, Dillan David Zachary Saunders, Lynn McTeir, Hazel Gilhooley, Melany Jackson, James D Glover, Jonathan Smith, Jeffrey J Schoenebeck, Laura A Lettice, Helen M Sang, and Megan G Davey

I have now received all the referees reports on the above manuscript, and have reached a decision. The referees' comments are appended below, or you can access them online: please go to BenchPress and click on the 'Manuscripts with Decisions' queue in the Author Area.

The overall evaluation is positive and we would like to publish a revised manuscript in Development, provided that the referees' comments can be satisfactorily addressed. In particular I would draw your attention to the comments from Referee 3 concerning the summary Figure 6 and the role of Shh in patterning or cell survival.

Please attend to all of the reviewers' comments in your revised manuscript and detail them in your point-by-point response. If you do not agree with any of their criticisms or suggestions explain clearly why this is so. If it would be helpful, you are welcome to contact us to discuss your revision in greater detail. Please send us a point-by-point response indicating your plans for addressing the referees' comments, and we will look over this and provide further guidance.

Reviewer 1

Advance summary and potential significance to field

unchanged since original submission

Comments for the author

The authors have addressed all my comments.

Reviewer 2

Advance summary and potential significance to field

The authors extend our knowledge on the cellular and genetic networks involved in the development of the zeugopod bones (radius, ulna, tibia, and fibula), particularly focusing on the contribution of SHH-expressing cells and differences between chicken and mouse ulna development.

Comments for the author

© 2024. Published by The Company of Biologists under the terms of the Creative Commons Attribution License (https://creativecommons.org/licenses/by/4.0/).

I acknowledge that the authors have addressed the majority of my comments.
However, two significant points remain unaddressed. Firstly, the analysis of the ZPA contribution to the fibula in mice. This entails lineage marking in mice, and it is puzzling why the authors did not retrieve tissue for this purpose. I wonder whether the authors could provide an explanation for this in their paper, as it would inform other researchers of potential difficulties of this approach.
Secondly, I miss some discussion on long-range versus short-range Shh signaling in chick versus mouse (as study delineates an extensive area expressing Ptc1 (a Shh target) but lacking Shh expression).

Reviewer 3

Advance summary and potential significance to field

This paper highlights a new method for very precise lineage analysis in chick.
Results clearly demonstrate the utility of direct TAT-Cre application in ovo used in conjunction with a chick line transgenic for conditional fluorescent reporters. The technique is novel and has the potential for much greater precision in lineage tracing than achieved with either focal dye labeling in the chick model, or using tissue-specific Cre drivers in mouse. They apply this tool to evaluate the contribution of the Shh-descendant lineage to posterior long bones and demonstrate striking differences in contribution to the homologous fore- and hindlimb bones (ulna, fibula) as well as differences with mouse, which has implications for models of primary limb axis evolution.

Comments for the author

Overall, the revised manuscript is greatly improved and the writing is more concise and clearer. In particular, the additional detailed information on the novel lineage tracing approach is informative and will be of great interest. I have a couple of minor comments (listed below) regarding the new figures included that the authors should take note of.
I do have one more serious concern about the impression conveyed by the summary cartoons in Figure 6 together with numerous statements in the text that zeugopod and digits 2,3 are "patterned" or "specified" by paracrine Shh signaling.
Clearly, they all respond to paracrine (long-range) signaling (at least by HH20), but whether this response plays a role in specification, patterning or in promoting cell survival and growth cannot be ascertained from lineage tracing or HCR expression analyses. At least in mouse hindlimb development, the role of paracrine signaling has been shown to be critical for cell survival but dispensable for specification. Enforcing cell survival after very early complete removal of Shh activity (at a stage equivalent to chick HH17) rescues normal formation and morphology of all long bones and all digits (Zhu, 2022).

At this early stage, paracrine signaling is not observed. Chick may be different, but the experiments in this paper don't address it. I would urge the authors to modify their statements to avoid this very misleading impression and use less insinuating terms.

1. in Sup. Fig 5 – it appears that there is scattered weak expression of tdtTomato and EYFP seen to the far right of the image at 10hrs., at a seemingly distance that is quite far away from the TAT-Cre bead. Can this be clarified?

2. in Sup. Fig 8 – there is an error in the legend title (“topically” should be topical).
In the image shown, it also seems that the 4h bead exposure induces many more labeled cells than the 30sec exposure, but the 1hr and 2hr exposures do not (it actually looks like fewer induced than in the 30sec case). Is this just stochastic variation? Or an artifact of the focal plane shown? It is rather confusing without any explanation. Why not just show the 30sec and 4hr region?

Second revision

Author response to reviewers' comments

© 2024. Published by The Company of Biologists under the terms of the Creative Commons Attribution License (https://creativecommons.org/licenses/by/4.0/).

Reviewer 1

Reviewer 1 Advance Summary and Potential Significance to Field:
unchanged since original submission

Reviewer 1 Comments for the Author:
The authors have addressed all my comments.

Reviewer 2

Reviewer 2 Advance Summary and Potential Significance to Field:
The authors extend our knowledge on the cellular and genetic networks involved in the development of the zeugopod bones (radius, ulna, tibia, and fibula), particularly focusing on the contribution of SHH-expressing cells and differences between chicken and mouse ulna development.

Reviewer 2 Comments for the Author:
I acknowledge that the authors have addressed the majority of my comments.
However, two significant points remain unaddressed. Firstly, the analysis of the ZPA contribution to the fibula in mice. This entails lineage marking in mice, and it is puzzling why the authors did not retrieve tissue for this purpose. I wonder whether the authors could provide an explanation for this in their paper, as it would inform other researchers of potential difficulties of this approach.

Response: We have added further details about the mouse fibula (Lines 255-261), which we hope makes clear that for our own technical reasons we were unable to analyse this and so cannot comment on it.

In short, since the retiral of Professor Bob Hill, who maintained these mouse lines, we had very few transgenic mice. At the time of the analysis, we had only one ageing $Shh^{tm1(EGFP/cre)Cjt}$ male and few female Cre-inducible tdRFP mice which we could breed from. Most of our crosses were unsuccessful, probably because of the age of the male. Initially we were only looking to compare the chicken wing maps with location of SHH descendants in the forelimb and this is what we examined, although we did keep three mouse hindlimbs in 4% PFA. Only many months later, when we had discovered the difference in SHH-expressing cell contribution to the wings and legs in the chicken did we try to look at dtTom expression in these limbs. We tried with various methods to visualise the tdRFP expression in the mouse legs, but nothing we did worked, including using sectioning and using an anti-RFP antibody. The forelimb had already been difficult and that was relatively fresh tissue. We have been unable to generate any more mouse tissue since. We will add that we do think that looking at the distribution of Shh-expressing descendant cells in the mouse zeugopod would be interesting and would complement work by Susan Mackem's group which has looked at similar cell lineages but predominantly in the wholemounts of the hindlimb autopod. We unfortunately are not well placed with our resources to do this work.

Secondly, I miss some discussion on long-range versus short-range Shh signaling in chick versus mouse (as study delineates an extensive area expressing Ptc1 (a Shh target) but lacking Shh expression).

Response: We have added to the Discussion between lines 325-338, comparing long-range and short range contributions of SHH signaling in the chicken wing, leg and mouse forelimb.

Reviewer 3

Reviewer 3 Advance Summary and Potential Significance to Field:
This paper highlights a new method for very precise lineage analysis in chick. Results clearly demonstrate the utility of direct TAT-Cre application in ovo, used in conjunction with a chick line transgenic for conditional fluorescent reporters. The technique is novel and has the potential for

© 2024. Published by The Company of Biologists under the terms of the Creative Commons Attribution License (https://creativecommons.org/licenses/by/4.0/).

much greater precision in lineage tracing than achieved with either focal dye labeling in the chick model, or using tissue-specific Cre drivers in mouse. They apply this tool to evaluate the contribution of the Shh-descendant lineage to posterior long bones and demonstrate striking differences in contribution to the homologous fore- and hindlimb bones (ulna, fibula) as well as differences with mouse, which has implications for models of primary limb axis evolution.

Reviewer 3 Comments for the Author:
Overall, the revised manuscript is greatly improved and the writing is more concise and clearer. In particular, the additional detailed information on the novel lineage tracing approach is informative and will be of great interest. I have a couple of minor comments (listed below) regarding the new figures included that the authors should take note of.

I do have one more serious concern about the impression conveyed by the summary cartoons in Figure 6 together with numerous statements in the text that zeugopod and digits 2,3 are "patterned" or "specified" by paracrine Shh signaling.
Clearly, they all respond to paracrine (long-range) signaling (at least by HH20), but whether this response plays a role in specification, patterning or in promoting cell survival and growth cannot be ascertained from lineage tracing or HCR expression analyses. At least in mouse hindlimb development, the role of paracrine signaling has been shown to be critical for cell survival but dispensable for specification. Enforcing cell survival after very early complete removal of Shh activity (at a stage equivalent to chick HH17) rescues normal formation and morphology of all long bones and all digits (Zhu, 2022).
At this early stage, paracrine signaling is not observed. Chick may be different, but the experiments in this paper don't address it. I would urge the authors to modify their statements to avoid this very misleading impression and use less insinuating terms.

Response: The reviewer is correct in pointing out that we have over stated the case of SHH ‘patterning’ digits. This was not intentional and we have removed all statements about SHH specifying identity and have only used the word ‘patterning’ in a more generic context- not directly about the action of SHH itself (lines 63-64, lines 83-84).

However, the point of this paper is to examine how and if we can understand the identity of limb bones by the lineage of cells that contribute to them in the normal anatomy. In this case, we think the cartoon is important as it conveys, in the normal limb, what the established model is for what tissues are derived from a SHH expressing lineage (i.e. ZPA short range) Vs. cells subject to long range SHH signaling, alongside what we have shown. This then relates to the interpretation of the Primary Axis Model, digit loss and how the articulation between the zeugopod and autopod is evolutionarily flexible. We do think this adds to previous literature; for example in Figure 6 of Zhu et al. 2022 (https://doi.org/10.1016/j.devcel.2022.07.016) the entire zeugopod bone is coloured as “specified directly?”. We have been able to answer this somewhat- at least by showing that not all cells in the posterior zeugopod bone are from the same SHH expressing lineage.

We have modified the annotation of Figure 6, however, and would like to stress that we are not commenting on specification of digits, direct, indirect, relay or otherwise and there is no mention of this in the Figure legend.

5. in Sup. Fig 5 – it appears that there is scattered weak expression of tdtTomato and EYFP seen to the far right of the image at 10hrs., at a seemingly distance that is quite far away from the TAT-Cre bead. Can this be clarified?

Response: The fluorescent data we are commenting on in this figure (time of expression of tdTom and eYFP after TAT-Cre application) is the widespread expression, not the expression around the bead. This was an experiment we undertook soon after the line was generated when we could only undertake time-lapse imaging on one embryo at a time, blind without genotyping. To select an embryo for imaging and to allow the correct image settings, we placed a TAT-Cre loaded bead in several ex-ovo cultures, and examined embryos for localised fluorescence after 12 hours. We then applied widespread TAT-Cre protein across the embryo to maximise our chances of observing any fluorescence. To make this clearer we have changed the Supplemental Data slightly from *“Ex ovo EC culture*

© 2024. Published by The Company of Biologists under the terms of the Creative Commons Attribution License (https://creativecommons.org/licenses/by/4.0/).

Chameleon embryo imaged on a Nikon Eclipse Ti with mercury bulb illumination source, on which it was possible to observe only tdTom and mEYFP2. A bead soaked in TAT-Cre had previously been applied to the embryo (arrow) to induce tdTom and mEYFP2 in order to allow the correct image capture settings (i.e. exposure) to capture expression of newly induced tdTom and mEYFP2. Weak tdTom and mEYFP2 can be observed at 5 hours, which become stronger over time."

To

"Ex ovo EC culture Chameleon embryo imaged on a Nikon Eclipse Ti with mercury bulb illumination source, on which it was possible to observe only tdTom and mEYFP2. A bead soaked in TAT-Cre had previously been applied to the embryo (arrow) to induce tdTom and mEYFP2 in order to allow the correct image capture settings (i.e. exposure) to capture expression of newly induced tdTom and mEYFP2 which is observed widely to the right of the image. Weak tdTom and mEYFP2 can be observed at 6 hours, which become stronger over time."

6. in Sup. Fig 8 – there is an error in the legend title ("topically" should be topical).

Response: Many thanks, we have corrected this to 'topical'.

In the image shown, it also seems that the 4h bead exposure induces many more labeled cells than the 30sec exposure, but the 1hr and 2hr exposures do not (it actually looks like fewer induced than in the 30sec case). Is this just stochastic variation? Or an artifact of the focal plane shown? It is rather confusing without any explanation. Why not just show the 30sec and 4hr region?

Response: We think the variation is due to the unequal application of TAT-Cre in EC culture. Perhaps the point to note here is that the 4hour time point includes two points- the right is the control where the bead has not been removed. There are more cells labelled where the bead was removed after 4 hours (left) than any places where a bead was placed and not removed in place (24 hours, right)

We hypothesise that this is due to excess TAT-Cre protein transferring with the bead. In short, when transferring a TAT-Cre loaded bead into a limb bud of a stage 20HH embryo in ovo, excess TAT-Cre is probably removed as the bead passes through fluids of the egg and then ectoderm. In EC culture, however, the bead and all the excess TAT-Cre is applied directly to the ectoderm or endoderm and as a result more free TAT-Cre protein is applied and there is more variation between applications (as demonstrated in this figure).

We have shown all the data for fullness of disclosure and although this is a confusing figure but have also acknowledged in the figure legend that it is not a particularly robust experiment. At this point we instead started testing the way topical TAT-Cre induced fluorescence in the limb bud.

As noted briefly in the paper (Line 180 "The duration and distance from bead, of cells newly expressing tdTom/mCeru/mEYFP2 differs between ex-ovo EC culture (Fig. S5-8) and limb bud application" and we have added a note in line 185-186, suggesting that researchers test the way in which topical application of TAT-Cre can be used in their system of interest.

In addition, we have found some formatting errors which we have remedied
Line 252- SHH reporter mouse Shhtm1(EGFP/cre)Cjt to Shh reporter mouse $Shh^{tm1(EGFP/cre)Cjt}$
Line 503- SHH reporter mouse Shhtm1(EGFP/cre)Cjt to Shh reporter mouse $Shh^{tm1(EGFP/cre)Cjt}$
Line 837 - $SHH^{tm1(EGFP/cre)Cjt}$ to $Shh^{tm1(EGFP/cre)Cjt}$

Third decision letter

© 2024. Published by The Company of Biologists under the terms of the Creative Commons Attribution License (https://creativecommons.org/licenses/by/4.0/).

MS ID#: DEVELOP/2023/202340

MS TITLE: Insights into Digit Evolution from a Fate Map Study of the Forearm Using 'Chameleon', a New Transgenic Chicken Line

AUTHORS: Julia Dong Hwa Oh, Lu Freem, Dillan David Zachary Saunders, Lynn McTeir, Hazel Gilhooley, Melany Jackson, James D Glover, Jonathan Smith, Jeffrey J Schoenebeck, Laura A Lettice, Helen M Sang, and Megan G Davey
ARTICLE TYPE: Research Article

I am happy to tell you that your manuscript has been accepted for publication in Development, pending our standard ethics checks.

© 2024. Published by The Company of Biologists under the terms of the Creative Commons Attribution License (https://creativecommons.org/licenses/by/4.0/).
